# Virtual Pseudonym-Changing and Dynamic Grouping Policy for Privacy Preservation in VANETs

**DOI:** 10.3390/s21093077

**Published:** 2021-04-28

**Authors:** Ikram Ullah, Munam Ali Shah, Abid Khan, Carsten Maple, Abdul Waheed

**Affiliations:** 1 Department of Computer Science, COMSATS University Islamabad, Islamabad 45550, Pakistan; mshah@comsats.edu.pk (M.A.S.); gallian92@gmail.com (A.W.); 2 Department of Computer Science, Aberystwyth University, Aberystwyth SY23 3DB, UK; abk15@aber.ac.uk; 3 Secure Cyber Systems Research Group, WMG, University of Warwick, Coventry CV4 7AL, UK; CM@warwick.ac.uk

**Keywords:** vehicular network, location privacy, grouping, pseudonym-changing, anonymization, LBS

## Abstract

Location privacy is a critical problem in the vehicular communication networks. Vehicles broadcast their road status information to other entities in the network through beacon messages. The beacon message content consists of the vehicle ID, speed, direction, position, and other information. An adversary could use vehicle identity and positioning information to determine vehicle driver behavior and identity at different visited location spots. A pseudonym can be used instead of the vehicle ID to help in the vehicle location privacy. These pseudonyms should be changed in appropriate way to produce uncertainty for any adversary attempting to identify a vehicle at different locations. In the existing research literature, pseudonyms are changed during silent mode between neighbors. However, the use of a short silent period and the visibility of pseudonyms of direct neighbors provides a mechanism for an adversary to determine the identity of a target vehicle at specific locations. Moreover, privacy is provided to the driver, only within the RSU range; outside it, there is no privacy protection. In this research, we address the problem of location privacy in a highway scenario, where vehicles are traveling at high speeds with diverse traffic density. We propose a Dynamic Grouping and Virtual Pseudonym-Changing (DGVP) scheme for vehicle location privacy. Dynamic groups are formed based on similar status vehicles and cooperatively change pseudonyms. In the case of low traffic density, we use a virtual pseudonym update process. We formally present the model and specify the scheme through High-Level Petri Nets (HLPN). The simulation results indicate that the proposed method improves the anonymity set size and entropy, provides lower traceability, reduces impact on vehicular network applications, and has lower computation cost compared to existing research work.

## 1. Introduction

The recent development of Intelligent Transportation Systems (ITS) play a pivotal role in making the lives of citizens more safe and comfortable on the road. One of the main goals of ITS is to create efficient traffic flows [1,2]. ITS integrate information and communication technology for collecting and disseminating road traffic-related information and data. They  can improve road safety, reduce collision, minimize environmental pollution, and provide convenience and entertainment services to the vehicle user. It is seen as strategic priority for industry to implement these technologies in connected vehicles on the road network. The use of communicating vehicles will increase road safety and improve road traffic efficiency. For this purpose, the concept of Vehicular Ad-hoc Networks (VANETs) has been introduced, providing a communication channel between road entities such as vehicles and Road-Side Units (RSUs). VANETs allow the development of advanced traffic management services in the road network where driver behavior, traffic flow, and road status can be shared between vehicles and infrastructure in the locality [3,4].

The basic technological elements that compose vehicular networks are sensors, radar, cameras, On-Board Units (OBU), Event Data Recorders (EDR), Global Positioning System (GPS), and omnidirectional antennas [5]. An OBU facilitates communication between one vehicle and other vehicles and road infrastructure. An EDR records all the communication events that occurred during a vehicle trip. Precise location coordinates are accessed and updated with the help of GPS. Radar and sensors indicate various types of obstacles and dangerous situations on the road. A tamper-proof device can be installed in the OBU to protect confidential vehicle information from an outsider attacker. The communication models of VANETs are divided into two categories namely, Vehicle to Vehicle (V2V) and Vehicle to Infrastructure (V2I) [6,7]. While V2V provides communication between mobile entities, i.e., vehicles moving on the road, V2I supports communication between vehicles and infrastructure, exchanging various types of road environment information. Infrastructure is used as a gateway to the network authority. The communication technologies utilized include Dedicated Short-Range Communication (DSRC), WiMax, Cellular Network, WiFi and the VeMac protocol [8]. DSRC is one of the most widespread technologies used in automotive vehicles and is used as a standard of wireless communication technology in many cars in the market [9]. It is based on the IEEE 802.11p amendment of the IEEE 802.11 standard and operates in the dedicated frequency spectrum of 5.9 GHz.

VANETs applications can be divided into two categories, i.e., safety-related applications and comfort applications [1]. The main aim of safety-related applications is the safety of drivers and passengers through the dissemination upcoming hazardous events on the road networks. These applications provide protection by updating the current state of the surrounding environment of the road [10]. It includes road accident information, collision avoidance, lane changing warning, emergency, and so forth. Comfort or non-safety applications provide convenience and comfort to travelers on the road. This includes weather information, location services, nearest restaurant, games, advertisements, and so on. The Basic Safety Message (BSM) or Cooperative Aware Message (CAM), are beacon messages that are broadcast through the road environment to inform other nodes in the network about the road status information. The message contains the vehicle identity, speed, direction, location, and other information. This information is disseminated in the network in an open form, and everyone can access it, even if they are adversary or attacker. If an adversary catches the actual identity of a vehicle during a trip, they may come to know various locations visited by the vehicle driver. In this way, the adversary comes to know the behavior of the vehicle driver, producing various types of threats that may be physical harassment, damage to social reputation, property loss, and blackmail [11].

To protect location privacy in the case of VANETs, a pseudonym may be used in the beacon message instead of the actual identity of a vehicle. A pseudonym is a temporary identifier often used for a short period of time. However, the use of fixed pseudonyms don’t solve the privacy problem of a vehicle driver. Pseudonyms should be changed periodically to confuse an adversary about the actual vehicle communicating during a road network journey. To solve the problem of location privacy in existing literature, various pseudonym-changing schemes have been proposed that are based on the concept of a mix zone concept [12,13,14,15] and a silent period [16,17,18]. In the mix zone method, vehicles’ identities are mixed in specific areas that may be a fixed zone at a road intersection or some congested area of vehicles. These schemes can achieve a high level of location privacy in high vehicle traffic and at selected places. Still, they have certain limitations that make it difficult to provide privacy in lower-traffic conditions. The vehicles remain silent by not broadcasting beacons messages in silent period-based schemes. This hides the identities of a vehicle for a certain amount of time. These techniques have specific limitations; for example, the achievement of privacy at the cost of compromising road safety application of the vehicular network. There are some situations in which it is difficult for a vehicle to become silent in a cooperative manner; ultimately, the vehicle should remain silent in an independent manner, which reduces privacy protection. The schemes mentioned above are challenging to apply in the case of higher speed travel and low traffic density. There remains a need for a scheme that provides location privacy on a highway or main roads where vehicles are moving at high speed. To solve the problems mentioned in the existing literature, we propose a new scheme of distributed and dynamic virtual grouping that protects location privacy on main roads of the vehicular network.

The context-aware scheme CLPS, ref. [18] requires a vehicle to be synchronized with silent neighbors to change pseudonyms during a silent period for identity protection. However, the using isolated roads and short silent periods can assist an adversary in detecting the target vehicle. Moreover, once a subject vehicle has knowledge of neighbor’s pseudonyms, which provides a way for a linkability of pseudonyms. In [10], privacy is also preserved only while the vehicle is in the communication range of RSU.

In this paper, we propose a Dynamic Grouping and Virtual Pseudonym (DGVP) exchange scheme that provides location privacy to the vehicle in the road network. Our contributions in this paper are given below.

- We introduce the concept of distributed and dynamic grouping based on road context information. Vehicles with similar status are combined in a group whose size depends on the number of transmission range neighbors; the pseudonyms are changed cooperatively.
- The proposed scheme improves location privacy in low vehicle traffic density environments. For this purpose, a virtual pseudonym update scheme is used, in which vehicles generate some randomized version of a pseudonym to create uncertainty for an adversary in recognizing a target vehicle in a region of interest.
- We also considered the privacy protection mechanism for vehicle communication with LBS in which a vehicle requests the nearest location. In this case, we introduce a position-mixing method that mixes the positions of vehicles in the vicinity to protect vehicle identities.
- The proposed scheme DGVP uses road network information and does not change any road status information in beacon messages, which would reduce the privacy scheme impact on VANETs applications.

The remainder of the paper is organized as follows. Section 2 contains a literature review. Section 3 has detail information about system models and goals. The proposed scheme is discussed in Section 4. Formal modeling and specification are given in Section 5. In Section 6, the experimental evaluation is discussed in detail. Section 7 contains the performance comparison of the proposed scheme. The position-mixing method is analyzed in Section 8. Analysis and discussion are specified in Section 9, and finally, the paper is concluded with Section 10.

## 2. Related Work

**Figure 1:**
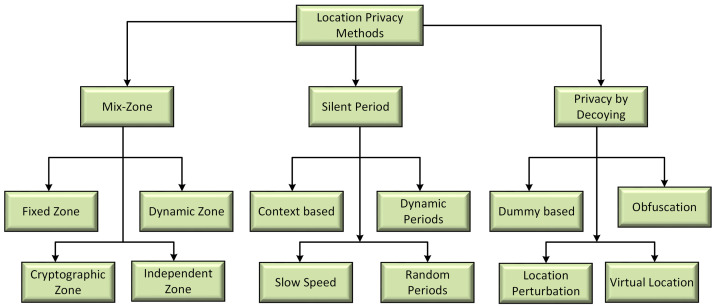
Location privacy protection techniques taxonomy.

This section contains a review of some of the existing privacy protection schemes in the literature. A pseudonym is used in a number of existing privacy schemes instead of the actual vehicle identity in the beacon message. The vehicle broadcasts beacons, which contain a pseudonym that anonymizes the vehicle. However, when using a fixed identifier (pseudonym) in a beacon message for a vehicle trip a privacy danger arises. An adversary can link the pseudonyms of a vehicle with the various locations visited. To protect a vehicle’s privacy, a pseudonym must be periodically changed to prevent an adversary from locating a particular vehicle on the road network. There are several pseudonym changing schemes in the literature. Table 1 contains a comparative analysis of existing location privacy schemes. The detailed taxonomy diagram of location privacy techniques is shown in Figure 1. Some of the existing privacy-preserving strategies are discussed below.

The cryptographic mix zone is established at a road-side intersection in [19], at which vehicles change temporary identifiers. The zone is combined with a mix network to provide pseudonym unlinkability. The vehicles in fixed zones are forced to change pseudonyms. The extended cryptographic zone scheme is proposed in [20] to tackle vulnerability to internal attackers. The RSUs are involved in the pseudonym-changing process. Similarly, in [21], mix zones are created in social spots such as parking lot. The vehicles that are gathered at social places change pseudonyms collectively to hide location information. The concept of a dynamic mix zone is introduced in [22], based on vehicles’ requests. Traffic statistics and privacy requirements determine the size of the zone. The messages communicated in the zone are encrypted to avoid pseudonym linkability. Similarly, in [23], the vehicles form a mix zone dynamically and motivate the neighboring vehicles to take part in the pseudonym change process. A vehicle finds neighboring vehicles a similar status in [24] and exchange pseudonyms with neighbors based on trigger information, thereby confusing an adversary about the actual identity of a vehicle.

Qasim et al. introduced the concept of multiple mix zones [25,26], where the vehicles change pseudonyms dynamically. The pseudonym alteration is based on vehicle direction, displacement, and acceleration. Guo et al. proposed a scheme using an independent mix zone [13] where each vehicle creates some randomized version of pseudonyms to establish an anonymous zone of vehicles on the road network. In [12], fixed mixing zones are establish at road intersections. Vehicles change pseudonyms only at the fixed zone to protect location information under different traffic conditions. However, the scheme creates an extra cost of deployment and suffer from duplicated data. A new concept of creating a silent mix zone is presented in [27,28], where vehicles remain silent and exchanging their pseudonyms under the control of RSU. A distributed pseudonym-changing scheme is proposed in [29], also based on the construction of a silent mix zone. The zones are created based on vehicle traffic density. Location privacy strategies based on the mix zone concept have certain limitations. Firstly the cost of deploying zones at a road intersection requires infrastructure for implementation and of equipment installation, which can be costly. Secondly, in low vehicle traffic density it can be challenging to achieve the necessary level of privacy protection.

In [30,31], the concept of a silent period combined with group navigation is proposed for location privacy in a vehicular network. One of the vehicles is selected as a group leader (GL), and only the GL communicates with the network, while the remaining members of the group remain silent as a technique to hamper pseudonym linkability. Similarly, in [32], the vehicle remains silent under at lower speeds. When a vehicle is traveling at low speed, the probability of road accidents is lower. Based on this concept, a vehicle should keep silent and not broadcast beacon messages, to hamper pseudonyms linkage. A safe distance metric is introduced in [33] that searches radius for obfuscation in which the values of velocity, direction, and position are considered to hide vehicles’ identity. If a vehicle does not find any neighbor at a safe distance, then it remains silent for a random period. In [34], the local parameters of speed and direction are taken as input to update the pseudonym autonomously. The vehicle will change pseudonyms in silent mode to meet a certain traffic threshold; otherwise, it will wait for another cooperative silent period. A context-based scheme is introduced in [17] in which the vehicle entry and exit from a silent period is based on the number of neighboring vehicles and pseudonyms are changed cooperatively. Similarly, in [18], the vehicle waits for at least *k* silent neighbors before changing the pseudonym. Further, misbehaving vehicles are detected in the network and considered before changing the pseudonym. Another silent period scheme uses the concept of scheme permutation [16], in which vehicles exchange pseudonyms. This increases the confusion of an adversary about the actual location of a vehicle. Limitations of silent period location privacy schemes include that they affect road safety applications; the use of a short silence period provides a way for pseudonyms to be linked, while for a long silent period the adversary may find temporal and spatial relationships to track the location of a vehicle in the network [15].

A path confusion scheme is proposed in [35] that slightly changes the informed positions of two users in proximity. This decreases the probability of vehicle tracking for fixed adversary strength. In [36], to increase adversary confusion in linking pseudonyms of a vehicle, inaccurate beacons are added in between the accurate beacon messages. For using location services, the concept of decoy vehicles is used in [37], in an attempt to protect the location of other vehicles in the network. The decoy vehicle communicates with LBS on behalf of network vehicles. To break the continuous path with LBS in [38], vehicles cooperate to generate plausible locations for each other to conceal/obfuscate the actual location information. Similarly, in [39], the target vehicle takes the surrounding neighboring vehicle’s virtual location dynamically to mislead an adversary about the actual driver route on the road. In [40], a location privacy method based on blockchain is presented that satisfies the requirements of k-anonymity and does not rely on third party server. A route discovery protocol is presented in [41] that helps the source node to find an efficient path to a destination dynamically, and provides privacy protection to a node through a privacy-preserving verification process. A cache-based user location privacy protection scheme is introduced in [42] during communication with LBS. Limitations of using inaccurate data, introduced in these techniques is the impact on road network applications. In addition, extra overhead is introduced into the network due to duplicated data and redundant data.

**Table 1:** Comparative analysis of existing location privacy schemes.

Ref:	Execution Mode	Evaluation Metric	Procedure	Adversary Model	Accountability	Cost of Computation	Impact on Applications
[13]	Infrastructure less	Anonymity	Using dummy data	External global adversary	No	Reduced	Yes
[17]	Infrastructure less	ASS, entropy, traceability	Silent mode	Global passive adversary	No	Not calculated	Reduced
[43]	Infrastructure less	Protection rate	Cooperative	General adversary	No	Not given	Yes
[21]	Infrastructure-based	ASS, location privacy gain	Identity mixing	GPA	No	Reduced	Yes
[24]	Infrastructure less	Anonymity, entropy, tracking percentage	Triggered-based	External passive adversary	Yes	Not calculated	No
[33]	Infrastructure less	Anonymity, traceability	Silent mode	Global passive adversary	No	Not mentioned	No
[36]	Infrastructure-based	ASS, entropy, traceability	Route confusion	General attacker	No	Not mentioned	Yes
[44]	Infrastructure-based	ASS	Random selection	Passive adversary	No	Increased	Yes
[45]	Infrastructure less	ASS, entropy, tracking probability	Silent mode	Global passive adversary	No	Reduced	Yes
[18]	Infrastructure less	ASS, entropy, confusion, traceability	Scheme permutation	Global passive adversary	Yes	Not computed	No
[46]	Infrastructure-based	ASS, entropy, tracking probability	Cheating detection	Global passive adversary	No	Not mentioned	Yes
[47]	Infrastructure less	ASS, entropy, tracking probability	Use dummy data	Global passive adversary	No	Not computed	Yes

In the literature, much attention is given to the mix zone or silent period location privacy schemes; however, the mix zone concept only covers a limited area, and the vehicle privacy is protected only in the zone. In silent period location privacy techniques, vehicles do not broadcast beacon messages, and this can impact on road network applications, and sometimes vehicle change pseudonyms individually in particular road network cases (lower-traffic condition), which provides a way for an adversary to link the old pseudonym with the new one. There are some areas on the road network in which vehicles are moving at high speed and with lower traffic density such as highways or main roads affecting location privacy. Therefore, we propose a new dynamic grouping and virtual pseudonym-changing scheme that can be applied in such a road network scenario.

## 3. Models and Goals

This section contains the detail of models and goals. The models presented are the system model and the adversary model. The goals of the research paper are also presented and the vehicle registration process is discussed.

### 3.1. System Model

**Figure 2:**
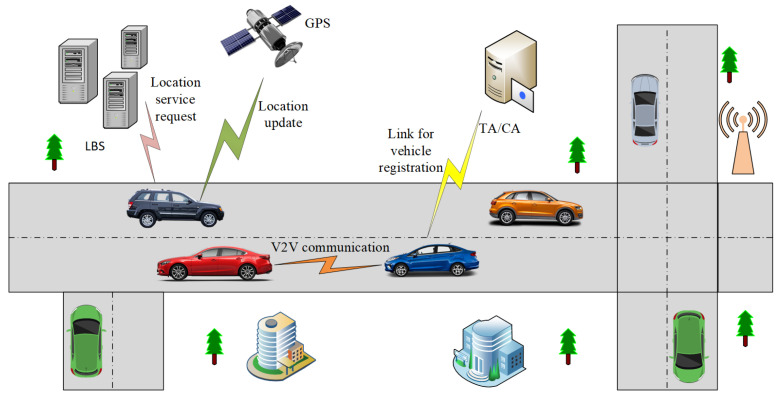
Basic system model.

Our system model consists of three entities: vehicle; Location Based Services (LBS); and Trusted Authority (TA). These are shown in Figure 2. TA is the registration authority that registers and provides certificates to the vehicles. It contains details of vehicle registration data and has a pseudonyms pool to assign to vehicles during registration. TA is considered trusted in our system model, does not compromise the privacy of vehicles. LBS is a location service provider that makes available various locations of interest to vehicles. For example, if a vehicle requires the nearest hospital or restaurant location, it can a request to LBS. However, LBS is not a trusted entity in the system model and can assist an adversary in compromising vehicles’ location. The third entity in the system model is the vehicle that is moving on the road, and our prime concern in this research is to provide location privacy to it. The vehicle is equipped with an OBU, which keeps a record of all events during a vehicle’s journey on the road network. The data in the OBU is given security with the help of a tamper-proof device. For the precise location data, the vehicle utilizes GPS.

### 3.2. Adversary Model

**Figure 3:**
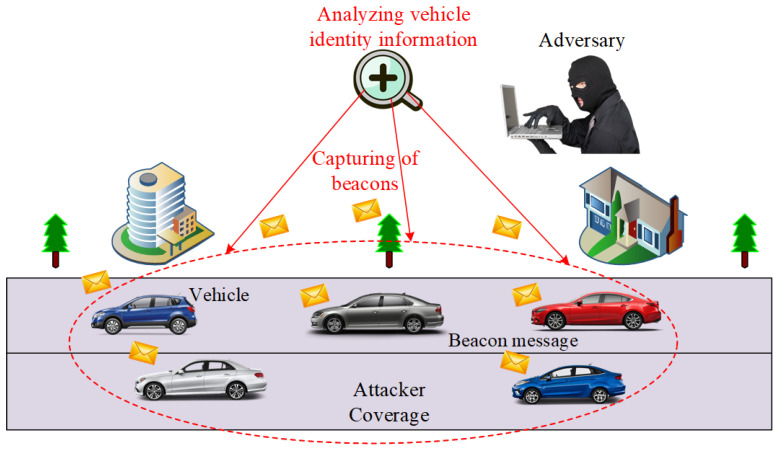
Adversary model.

In this research, we take a robust adversary model. It consists of an external Global Passive Adversary (GPA), which covers a large part of the network by deploying low-cost transceivers. Figure 3 shows the coverage area of the GPA, which analyzes the data collected from various vehicles moving on the road network. The GPA can eavesdrop on any messages communicated within its coverage [29]. The primary objective of the GPA is tracking or collecting vehicle location traces during a trip on the road using captured beacon messages [48]. The compromising of location privacy requires the de-anonymization of vehicle location tracks. The de-anonymization is successful if the attacker correctly reconstructs the location traces. The GPA tries to correlate the beacon messages of old and new pseudonyms for vehicle identification. The adversary tries to match the pseudonyms of vehicles at the various visited locations. We assume that the adversary can also obtain the location data of vehicles from LBS. Our scheme will provide protection both against GPA and LBS adversary.

The adversary can capture beacon messages broadcast during the vehicle’s journey on the road at the time. The beacon contains vehicle identity, position, and speed. In the proposed scheme, we use $G_{ID}$ instead of the pseudonym in the beacon message. The adversary captures the beacons of vehicles at time *t* and collects vehicles traces that contain information such as group identity, pseudonym, vehicle position and speed (S). Hence, the collected traces of data of different vehicles at time *t* is given by:

(1) $$\begin{array}{c} Tr=\sum_{i=1}^{n}(\left(G_{ID}\left(P_{ID}\right),POS,S\right)V_{i}. \end{array}$$

where Tr is the initial traces collected by an adversary, $G_{ID}$ is group identity, $P_{ID}$ is pseudo-identity of a vehicle, POS is the current position and S is the speed of a vehicle $V_{i}$. Over time the vehicle changes position and pseudonyms for privacy protection. At time $t^{\prime }$ the adversary collects another trace of various vehicles moving on the road that consists of vehicles pseudonyms, position and speed. The next set of traces $Tr^{\prime }$ collected by the adversary is given by the following equation:

(2) $$\begin{array}{c} Tr^{\prime }=\sum_{j=1}^{n}({\left(G_{ID}\left(P_{ID}\right),POS,S\right)}^{\prime }V_{j}. \end{array}$$

After collecting both traces, the GPA can analyze the vehicle data and try to identify the target vehicle. The traces of information contain only $G_{ID}$, location, and speed of the vehicle, where $P_{ID}$ is changed inside the group of vehicles. The following equation states the analysis process of GPA:

(3) $$\begin{array}{c} Analysis\left(GPA\right)=\sum_{i=1}^{n}Matching\left(Tr,Tr^{\prime }\right)V_{i}. \end{array}$$

Now the GPA will try to link pseudonyms of a target vehicle at various locations based on the vehicles collected traces at different periods. Here, the adversary takes probability Pr of matching the pseudo-identities of vehicle in the collected traces. The linking of vehicles pseudonyms at different locations is considered by the adversary and can be described as follows:

(4) $$\begin{array}{c} Analysis\left(P_{ID},POS\right)=\sum_{i=1}^{n}Pr[\left(\left(P_{ID},POS\right)t\to \left(P_{ID}^{\prime },POS^{\prime }\right)t^{\prime }\right)V_{i}] \end{array}$$

The vehicle communicates with LBS for the nearest location of interest. The location message contains group identity and vehicle location. The adversary captures the location data during this communication. The captured location data of vehicles by an adversary is given below:

(5) $$\begin{array}{c} Capture\left(LOC\right)=\sum_{i=1}^{n}MSG\left(G_{ID},LOC\right)V_{i}. \end{array}$$

After collecting information about different vehicles during communication with LBS, the adversary starts to analyze it. The adversary matches pseudo-identities of vehicles at different locations and tries to extract a target vehicle’s actual identity and locations. The overall prediction of the GPA for identifying the target vehicle is stated in the following equation.

(6) $$\begin{array}{c} Analysis\left(Overall\right)=\sum_{i=1}^{n}Pr[\left((G_{ID}\left(P_{ID},LOC\right)t\to G_{ID^{\prime }}\left(P_{ID^{\prime }},LOC^{\prime }\right)t^{\prime }\right)V_{i}]. \end{array}$$

### 3.3. Goals

The main concern of this article is to protect the location privacy of vehicles in a vehicular network. We set the following goals in this research work.

1. Construction of dynamic grouping of vehicles at diverse nature of road network.
2. Virtual pseudonym change scheme in case of lower vehicle traffic.
3. Protection of a vehicle location information while querying to LBS.
4. Reduce the impact of privacy on VANETs applications.
5. Create uncertainty for an adversary to link the pseudonyms of a vehicle at different locations.

### 3.4. Vehicle Registration

Before, initial road network deployment vehicles must register with a Trusted Authority (TA). The TA is a governmental authority that provides certificates to the vehicles at the time of registration. The vehicle registration is shown in Algorithm 1. First, the vehicle will request from TA the registration that binds the vehicle to vehicle identity, License Plate Number (LPN), and other necessary items. Upon successfully verifying LPN, the TA issues certificates to the requesting vehicle; otherwise, the invalid LPN vehicle request is rejected. The TA provides a set of *P* pseudonyms $PU_{i,k}$ to the vehicles, where *k* belongs to (1...P) that are public-key certificates. The beacon message is signed with a private key of the sender’s vehicle in connection with pseudonyms for proper authentication. When a message is broadcast by a vehicle in a region to disseminate road status information, upon reception of the message, the receiver vehicles in the vicinity can verify a sender’s vehicle authenticity with its public key [46,49]. The pseudonyms are used for a short period to protect the identity of vehicles. Pseudonyms have an expiry time, and after the expiration, the vehicle may request another pseudonym pool. In our case, we set a pseudonym pool for long periods, i.e., for a number of days (week/month).

**Algorithm 1** Vehicle Registration.**Initialization:**Vehicle(i): Any vehicle *i* request for registration, $V_{ID}$: Vehicle identity, LPN: License Plate Number, PseudoID: Pseudonym identity of a vehicle
Expiry(PseudoID): The vehicle monitors pseudonyms pool expiry, $Valid(LPN_{i})$: Validity of vehicle LNP is checked, $Issuence(PU_{i,k},P)$: issue certificate with pseudonym pool **Input:**
$V_{ID},LPN$ **Output:** Issuance of Certificate to vehicle
  1:**for**Vehicle(i)=1→n**do**  2:    $Request_{i}\left(V_{ID},LPN\right)\;\to \;TA$  3:    TA Verify $\left(LPN_{i}\right)$  4:    **if** $Valid(LPN_{i})$ **then**  5:           $Issuence(PU_{i,k},P)$  6:    **else**  7:           Discard ($Request_{i}$)  8:    **end if**  9:    Check Expiry(PseudoID)10:    **if** Expire(PseudoID) **then**11:           Go to step 212:    **else**13:           Continue Usage of certificate14:    **end if**15:**end for**


## 4. Proposed Solution

**Figure 4:**
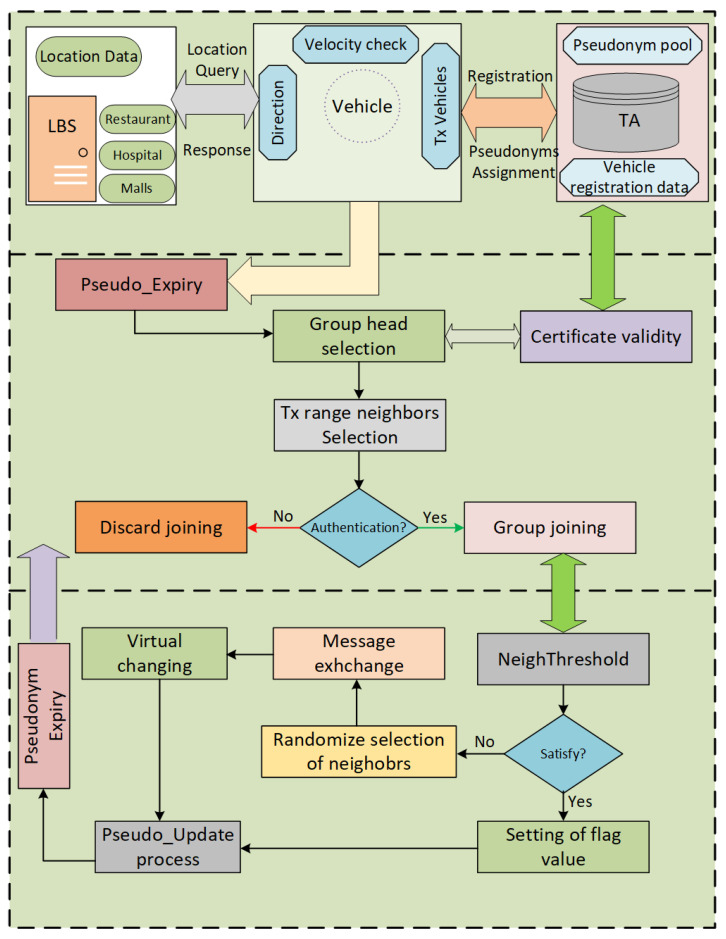
The block diagram of the proposed scheme.

This section contains the detail of the proposed scheme. It is a group-based location privacy scheme in the case of a vehicular network. There are some road network scenarios where vehicles have high speed and may have lower or higher traffic conditions. We proposed a new Dynamic Grouping and Virtual Pseudonym (DGVP) changing scheme for location privacy preservation. The proposed scheme is adaptive that considers the number of vehicles in transmission range. We consider also the velocity range, to make a group of vehicles to change the pseudonyms cooperatively. One of the vehicles is selected randomly as Group Head (GH) that will monitor all the vehicles’ change of pseudonyms in the group. This kind of situation usually occurs on a main road where vehicles are moving at high speed. The group of vehicles is formed based on similar velocity range and same direction. The block diagram of the proposed scheme is shown in Figure 4. The GH collects information about transmission range vehicles and allows them to join the group. After that, the pseudonym change process is initiated. Pseudonyms of vehicles in the group are changed based on the number of neighboring vehicles. The neighbor threshold is used to apply the cooperative pseudonym update process or the virtual pseudonym update process. The proposed scheme’s main components are vehicle grouping, pseudonym-changing protocol, and vehicle to LBS communication.

### 4.1. Vehicle Grouping

**Figure 5:**
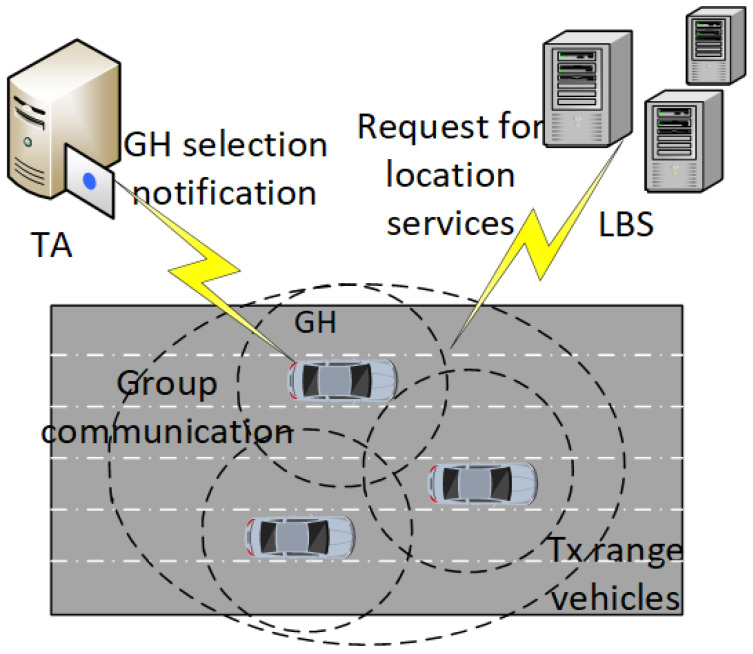
Selection of GH and group communication.

Once a GH has been selected, its identity is verified with the help of the certificate provided at the time of registration. The selection of GH vehicle detail is given in Algorithm 2. The GH-selection notification and group communication are shown with the help of Figure 5. The GH selection is announced in the vicinity, and the transmission range vehicles request to GH to join the group. Before joining the group, GH authenticates vehicles through a signature scheme (certificates assigned to vehicles by TA). GH prohibits the joining of any vehicle with an invalid certificate. In this way, vehicles with malicious intent could not join the group. A group identity ($G_{ID}$) is created and distributed through the group. All the communication in the group is verified with $G_{ID}$. The GH informs the members of the group about the pseudonym change process. For this purpose, a flag is set to 1, which means ready for pseudonym change. When all the group members change pseudonyms, and the flag is set to 0, the pseudonym is changed successfully. The detail of the vehicle group formation protocol is given in Algorithm 3. First, the vehicles in the transmission range identified. Next, the signature verification process is started for vehicles joining in the group. Out of range vehicles will not take part in the group formation and communication process.

**Algorithm 2** Group Head Selection**Initialization**: Vehicle(i): Any vehicle on the road, PseudoExpiry(i):Pseudonym expiration of vehicle *i*, GH: Group Head, SendGHselction(i): Send vehicle *i* to TA for GH selection **Input**: Vehicle pseudonym **Output**: Selection of GH
  1:**for**$V_{a}$∈vehicle(i)**do**  2:    PseudoExpiry(i)  3:    SendGHselction(i)→TA  4:    Verification Process  5:    **if** Vehicle(i)∈ValidCredential **then**  6:          Random selection of Vehicle(i) as GH  7:    **else**  8:          Discard  9:    **end if**10:**end for**11:Return(GH)


**Algorithm 3** Vehicle Grouping**Initialization**: $V_{i}$: Any vehicle *i* moving on the road, GH: Group Head, $T_{x}$: Transmission range, $G_{ID}$: Group identification, $VL_{R}$: Vehicle velocity range, *D*: Direction, $PseudoExpiry(V_{i})$: Vehicle pseudo id expiration, Verification(GH): Verification of group head, NeighvehicleRange(i): Neighbor vehicle *i* is in transmission range, $Sign(V_{i})$: Signature of vehicle *i*, Valid(C): Certificate validity, Group $\left(V_{i}\right)$: Grouping of vehicle *i*, Discard-joining $\left(V_{i}\right)$: Discard-joining of vehicle *i* in the group **Input**: $VL_{R}$, $T_{x}$, *D* **Output**: Group formation and communication process
  1:**for** all $V_{i}=1\to n$ **do**  2:      Check $PseudoExpiry(V_{i})$  3:      GroupHead()  4:      Set flag to 1  5:      Verification(GH)  6:      NeighborFunction()  7:      **for** each $Vehicle\left(i\right)\in VL_{R}$ **do**  8:            **if** $NeighvehicleRange\left(i\right)\leq T_{x}$ **then**  9:                 Process of authentication10:                 **if** $Sign\left(V_{i}\right)\in Valid\left(C\right)$ **then**11:                       GH allows joining $V_{i}$12:                       GH distribute $G_{ID}$ to $V_{i}$13:                       Group $\left(V_{i}\right)$14:                 **else**15:                       Invalid-certificate16:                       Discard-joining $\left(V_{i}\right)$17:                 **end if**18:            **else**19:                 Out of range20:            **end if**21:      **end for**22:      Return (Members)23:      Ready for Pseudonym change process24:**end for**


### 4.2. Pseudonym-Changing Protocol

**Figure 6:**
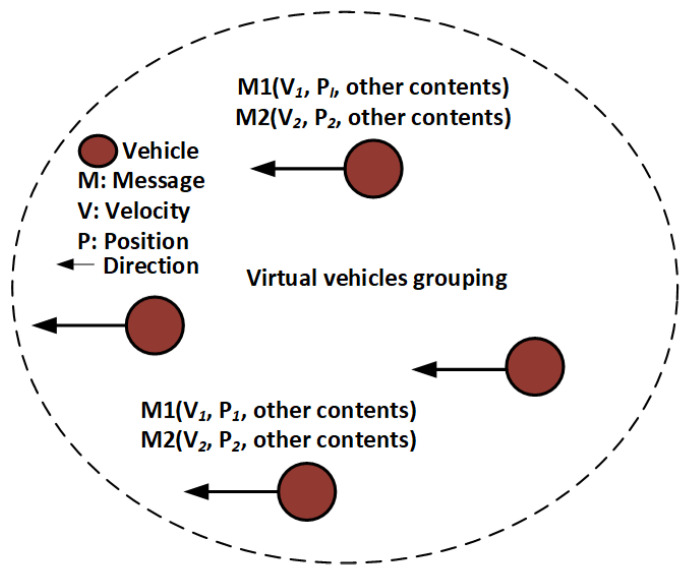
Virtual pseudonyms changing in the grouping concept.

In this section, we discuss the pseudonym-changing protocol of vehicles on the road network. The GH will monitor the number of neighboring vehicles in the group. A threshold is set for the maximum number of neighboring vehicles to change pseudonyms using road context information. The road context information includes vehicle velocity range, transmission ranges, number of neighbors in the vicinity. First, the neighbor function is applied to check the number of transmission range neighbors. After that, the neighbor threshold is verified before executing the pseudonym-changing process. Suppose the number of neighbors is greater than or equal to a threshold, then the GH disseminates information in the group to change pseudonyms cooperatively. Otherwise, the GH will announce the virtual pseudonym change process that is shown in Figure 6. The GH will select a few vehicles for a virtual pseudonym change. The chosen vehicles generate duplicate pseudonyms in the group and create a crowd of virtual vehicles in the vicinity. Algorithm 4 contains the detail of the pseudonym-changing protocol. The pseudonym-changing protocol is divided into two sections, i.e., the normal pseudonym update process and the virtual pseudonym update process.

**Algorithm 4** Pseudonym-Changing Protocol**Initialization**: Vi: Any vehicle *i*, NeighCount: Number of neighboring vehicles, NeighThreshold: Threshold for several transmission range vehicles, $T_{x}$: Vehicle transmission range, $VL_{R}$: Velocity range, $Pseudo-Update(V_{i})$: Change the pseudonyms of vehicle *i*, $Virtualizer(V_{i})$: Vehicle *i* take part in virtual pseudonym change process, PseudonExpiry(t): Pseudonym expiry time, NF: Neighbor Function, $VerifyAuthenticity(V_{i})$: Verification of vehicle *i* authenticity, Calculate Distance $\left(GH,V_{i}\right)$: distance calculation between GH and vehicle *i*, $DistanceV_{i}\left(minimum\right)$: Vehicle with minimum distance with GH **Input**: $VL_{R},T_{x},NeighThreshold$ **Output**: Successful change of pseudonym and anonymization in the group
  1:**for** all $V_{i}=1\in VelocityRange$ **do**  2:      $NF(VL_{R},T_{x},G_{ID})$  3:      **if** NeighCount≥NeighThreshold **then**  4:            $VerifyAuthenticity(V_{i})$  5:            $Pseudo-Update(V_{i})$  6:            Set flag to 0  7:      **else**  8:            GH notify virtual change process in the group  9:            **for** Vi∈Group(GH) **do**10:                 Calculate Distance $\left(GH,V_{i}\right)$11:                 **if** $DistanceV_{i}\left(minimum\right)$ **then**12:                       Select $Virtualizer(V_{i})$13:                 **else**14:                       Go to step 1015:                 **end if**16:            **end for**17:            $Virtualizer(V_{i})$ Messages creation18:            $Msg_{1}\left(Pseudo-id_{1},VL_{1},POS_{1}\right)$19:            $Msg_{2}\left(Pseudo-id_{2},VL_{2},POS_{2}\right)$20:            $Pseudo-Update[V_{i}\left(Msg_{1},Msg_{2}\right)]$21:            Set flag to 022:      **end if**23:      PseudonExpiry(t)24:      Set flag to 1 and go to step 225:**end for**


The neighbor function is used to find the number of transmission range neighbors in the concerned region. The neighbor function is shown in Algorithm 5. Vehicles broadcast beacon messages for road status information. The messages are received by each transmission range vehicle. The algorithm takes velocity range, transmission range, and distance as input.

**Algorithm 5** Neighbor Function**Initialization:**$V_{i}$: any vehicle *i*, $T_{x}$: Transmission range, $SP_{R}$: Speed Range, *D*: Direction of a vehicle, $CountV_{ID}$: Counting of number of vehicles, $MessageReceived(M_{i})$: Receiving message from vehicle *i*, Check($V_{ID},D,SP_{R}$): Checking of vehicle identity, direction and speed range, Calculate Distance $\left(V_{i},V_{j}\right)$: Distance calculation between neighboring vehicles **Input:**$SP_{R}$, $T_{x}$, *D* **Output:** Number of transmission range vehicles ($CountV_{ID}$)
  1:**for**$V_{i}=1\to n$**do**  2:      $MessageReceived(M_{i})$  3:      Check($V_{ID},D,SP_{R}$)  4:      Calculate Distance $\left(V_{i},V_{j}\right)$  5:      **if** $\left(V_{ID}\neq V_{ID}\left(i\right)\;and\;Distance<=300m\right)$ **then**  6:            $CountV_{ID}++$  7:      **else**  8:            Check again(Limit)  9:      **end if**10:**end for**11:Return $\left(CountV_{ID}\right)$


#### 4.2.1. The Normal Pseudonym Update Process

**Figure 7:**
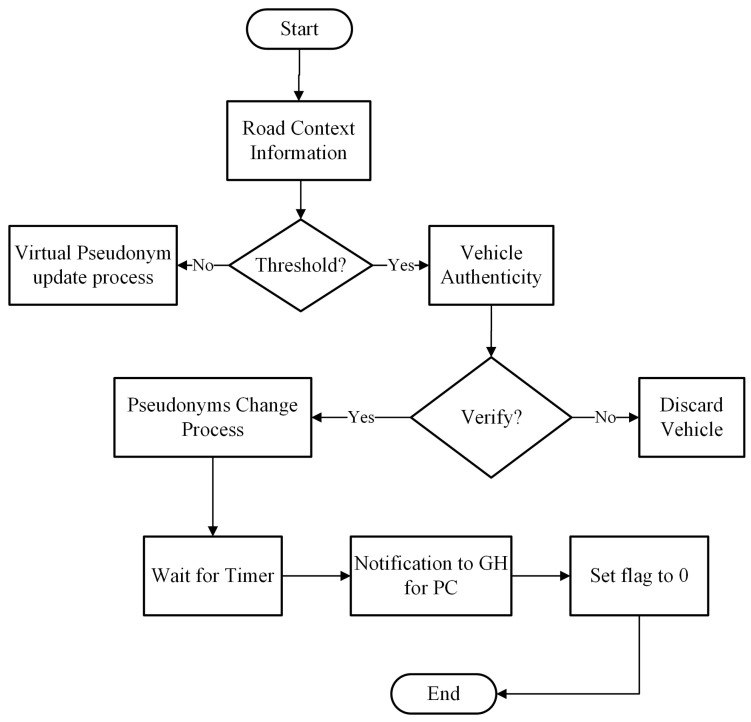
Flow of pseudonym update process.

The pseudonym update process takes road context information such as vehicle velocity range, direction, and transmission range neighbors. The GH will monitor the road environment for the change of the pseudonym process. The number of neighboring vehicles is calculated with the help of Algorithm 5. Once the NeighThreshold is met, the GH announces the group formation protocol for the collective pseudonyms change process as discussed in Section 4.1. Initially, each vehicle will set a flag value to 1, which means it is ready for the pseudonym updating process. A timer is used to trigger every member of a group to change pseudonyms simultaneously. After changing pseudonyms, the member vehicle notifies the GH and sets its flag value to 0, which means pseudonyms are successfully updated. The flow diagram of the pseudonyms update process at certain road context information is shown in Figure 7.

#### 4.2.2. The Virtual Pseudonym Update Process

**Figure 8:**
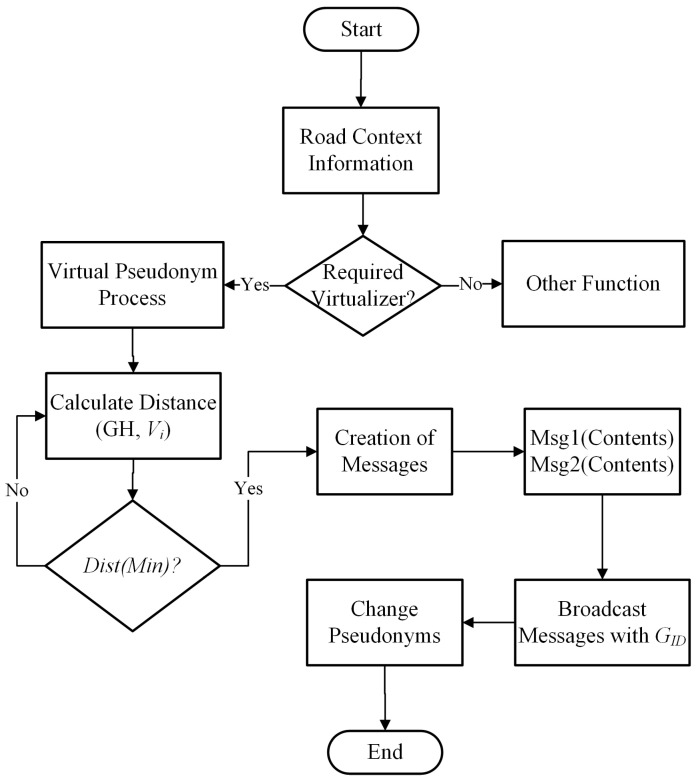
Virtual pseudonym update process.

The successful anonymization process of a target vehicle depends on the number of vehicles taking part in the pseudonym-changing process. If the vehicle traffic density does not fulfill the requirement for the protection of vehicles’ identity, there is a need for a suitable process that provides identity protection. We use a virtual pseudonym update process for the anonymization of vehicle identities in such a case. If the vehicle neighbor threshold is not satisfied, GH notifies the group’s virtual pseudonym change process. Each vehicle in the group will create two messages with different pseudo-IDs, velocities, and location positions. These messages are broadcast in the group with the same $G_{ID}$. This will hide the actual pseudonym of a vehicle during the grouping period. The virtual pseudonyms update process flow is shown in Figure 8.

### 4.3. LBS Communication

**Figure 9:**
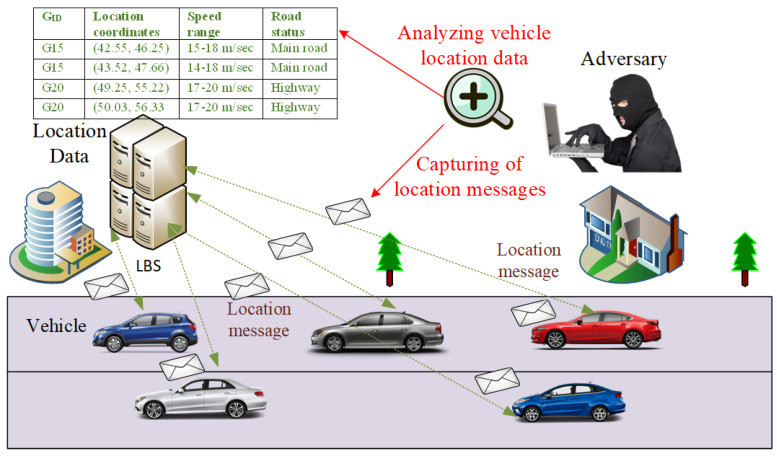
Adversary analyzing vehicle location data.

During a journey some vehicles will require the locations of places of interest such as the nearest shopping mall, restaurant, hospital, etc; a vehicle makes to request to an LBS. On obtaining a location query from the vehicle, the location service provider will respond to the concerned vehicle. In this research, LBS is not a trusted entity and may conspire with an adversary to compromise the location tracks of a vehicle. Figure 9 shows the adversary scenario capturing location data during vehicle communication with LBS. In this case, there is a need to safeguard the location traces of a vehicle. To protect the location’s traces of a vehicle in such a situation, we use a position-mixing procedure. The position-mixing algorithm requires two parameters, i.e., $G_{ID}$ and position coordinates of neighbors in the transmission range. The $G_{ID}$ hides the target vehicle’s actual identity. The vehicle will take the position coordinates of its neighbor randomly and will exchange its location position with it. The exchange of position coordinates mixes the target vehicle’s precise location with its neighbor. The basic position-mixing algorithm is given in Algorithm 6. First, the target vehicle will calculate the distance with each neighbor members of the group. A neighbor with a maximum distance range is selected for position coordinates exchange. For example, a vehicle $V_{i}$ finds another vehicle $V_{j}$, with a maximum distance range so that $V_{i}$ will take $V_{j}$ as a position mixer vehicle in the group.

**Algorithm 6** Position-Mixing Method**Initialization**: $V_{i}$: Any vehicle *i*, $G_{ID}$: Group identification, $POS_{i}$: Position coordinates of vehicle *i*, $POS_{i}$: Position coordinates of vehicle *i*, Distance(Max): Take neighbor vehicle with maximum distance range, $RandomSelection(V_{j})$: Random selection of any vehicle *j* as a position mixer, Exchange $\left(POS_{i},POS_{j}\right)$: Exchange of position coordinates between $T_{x}$ vehicles **Input**: $G_{ID}$, position coordinates **Output**: Mixing position coordinates
  1:**for**$V_{i}=1\to n$**do**  2:      NeighborFunction()  3:      Calculate $Distance(V_{i},V_{j})$  4:      **if** Distance(Max) **then**  5:            Select $\left(V_{j}\right)$ as a mixer  6:      **else**  7:            $RandomSelection(V_{j})$  8:      **end if**  9:      Take $V_{i}\left(G_{ID}\right)$10:      Exchange $\left(POS_{i},POS_{j}\right)$11:      Ready Message $\left(G_{ID},POS_{j}\right)$12:      Query $\left(G_{ID},POS_{j}\right)$ to LBS13:**end for**


**Figure 10:**
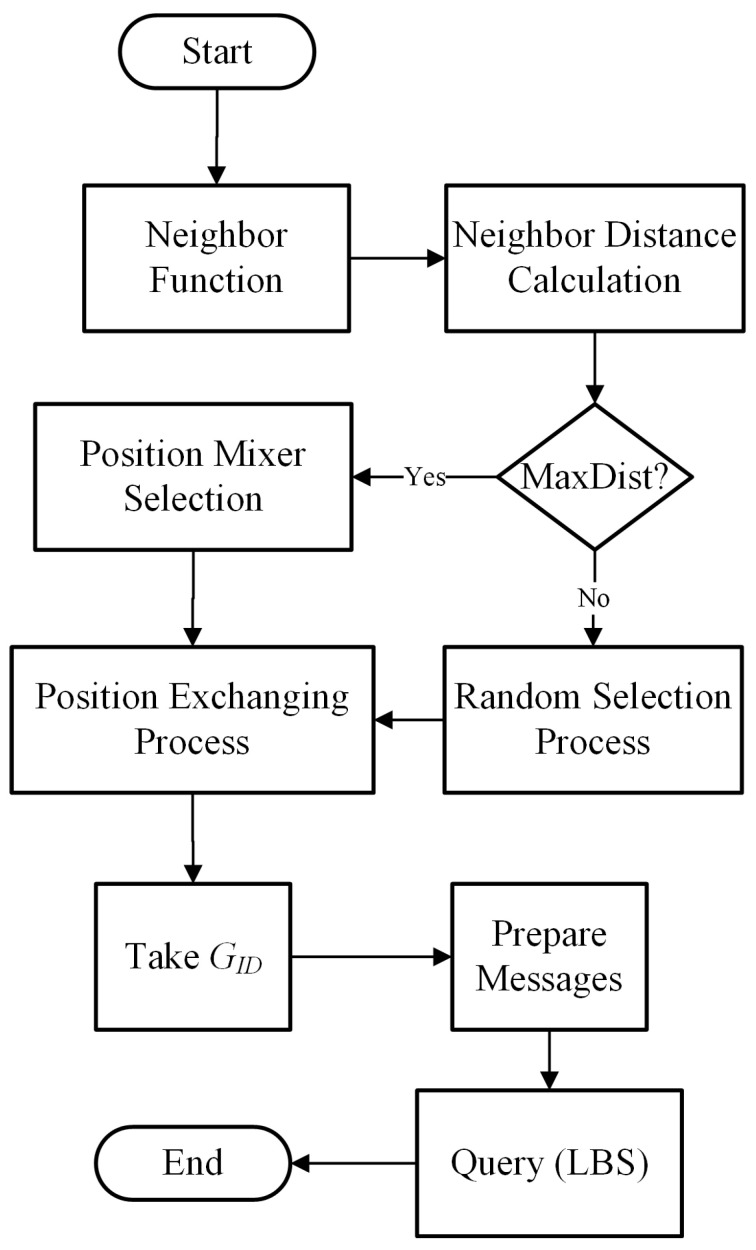
LBS query flow method.

The location position coordinates are exchanged between these two vehicles. Both the vehicles will send their queries with $G_{ID}$ and mixing positions to LBS for the nearest location of interest. On receiving location requests, the location provider will respond to each vehicle. The location request messages contain real position coordinates and the same group identities. Suppose someone (adversary) conspires with the location provider and wants to find a vehicle’s status; he/she (adversary) will find it challenging to identify a vehicle based on the position-mixing method because the location request message which contains a group identity that hides the actual vehicle and location coordinates of a neighboring vehicle protects the target vehicle’s location position. The flow procedure of query to LBS is shown with the help of Figure 10. If the distance ranges of a vehicle with neighbors are the same, it will randomly select one of these neighbors to participate in the position exchange process.

## 5. Formal Modeling and Specification

High-Level Petri Nets (HLPN) can be used for simulation of the proposed scheme or to provide a mathematical representation to analyze the behavior and structure properties of the proposed model [50]. The benefits of the formal model are the proposed model components and processes interconnections, information flow among the system processes, and information processing task are considered. HLPN consists of seven tuples that are:

1. *P* is a set of places.
2. *T* denotes a set of finite transitions such that (P∩T=∅).
3. *F* is a flow relation from place to transition and vice versa, i.e., F⊂(P×T)∪(T×P).
4. φ is a mapping function that maps places to data types such that φ:P→ Datatypes.
5. *R* denotes the set of rules that maps *T* to a logical formula, i.e., R:T→ Formula.
6. *L* presents the labels maps on each flow in *F* such that L:F→ Label.
7. $M_{0}$ denotes an initial state where the flow can be initialized, i.e., M:P→ Token.

In this section, we formally define and model the proposed algorithm, virtual grouping, and pseudonym-changing. Then we formally model and specify the attacker scenario on the DGVP scheme. In the third subsection, we design HLPN for the position-mixing algorithm and its attacker scenario.

### 5.1. Formal Modeling of Dynamic and Virtual Pseudonym-Changing Scheme

**Figure 11:**
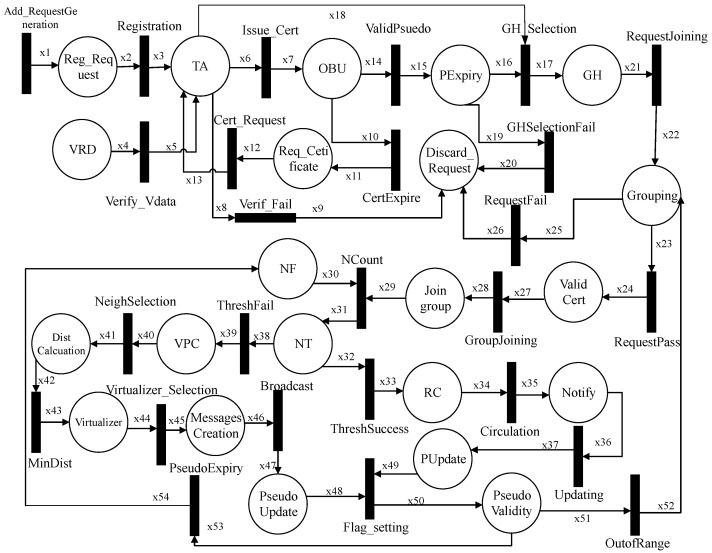
HLPN for DGVP scheme.

We formally define and analyze our proposed scheme DGVP in this section. The HLPN of the proposed DGVP scheme is shown in Figure 11, which contains details about the registration process, GH selection, vehicle grouping, and pseudonym-changing mechanisms. Table 2 includes a description of symbols used in the HLPN, while Table 3 describes the places used in the Petri nets.

Before joining the network, each vehicle must register with the Government Authority, i.e., TA. Each vehicle direct requests TA with their identity and LPN as given in Equation (7). TA verifies the vehicle data provided by the VRD authority in Equation (8). After the verification of a vehicle, TA provides certificates in Equation (9).

(7) $$\begin{array}{c} R(\mathrm{Registration})=\forall \;i2\in x2\wedge i3\in x3\;|\;i2[1]\neq i3[1]\wedge \\ x3^{\prime }:=x3\cup \left\{i2\left[1\right],i3\left[2\right],i3\left[3\right],i3\left[4\right]\right\}. \end{array}$$

(8) $$\begin{array}{c} R(\mathrm{Verify}-\mathrm{Vdata})=\forall \;i4\in x4\wedge i5\in x5\;|\;(i4[1]=i5[1]\wedge i4[2]=i5[2])\\ =Verified\to x5^{\prime }:=x5\cup \left\{i4\left[1\right],i4\left[2\right],i4\left[3\right]\right\}. \end{array}$$

(9) $$\begin{array}{c} R(\mathrm{Issue}-\mathrm{Cert})=\forall \;i6\in x6\wedge i7\in x7\;|\;Match(i6[1],i7[1])=True\\ \to x7^{\prime }:=x7\cup \left\{i7\left[2\right],i7\left[3\right],i7\left[4\right],i7\left[5\right]\right\}. \end{array}$$

In Equation (10), the TA discards the request of a vehicle with invalid credentials. The vehicle continuously checks the expiry of certificates. On the meeting certificate expiry condition as given in Equation (11), the vehicle requires another certificate because it cannot take part in communication in the network with an invalid certificate. For this purpose, the vehicle requests (Equation (12)) for another certificate.

(10) $$\begin{array}{c} R(\mathrm{Verify}-\mathrm{Fail})=\exists \;i8\in x8\wedge i9\in x9\;|\;(i8[1]\neq i9[1]\wedge i8[2]\neq i9[2])\\ =WrongCredentials\to x9^{\prime }:=x9\cup \left\{i9\left[3\right]\right\}. \end{array}$$

(11) $$\begin{array}{c} R(\mathrm{CertExpire})=\exists \;i10\in x10\wedge i11\in x11\;|\;(i10[1]=i11[1]\wedge \\ i10[4]=Expire)\to Required(\{i11[3]\}). \end{array}$$

(12) $$\begin{array}{c} R(\mathrm{CertRequest})=\exists \;i12\in x12\wedge i13\in x13\;|\;(i12[1]=i13[1]\wedge \\ Status(i12[4])=Required)\to Request(\{i13[1],i12[3],i12[4]\}). \end{array}$$

A pseudonym validity is checked in Equation (13); if a vehicle pseudonym has not reached its expiry period, it will continue to broadcast with a valid pseudonym. Otherwise, the vehicle will take another pseudonym from its pseudonym pool. For the change in pseudonym, the GH-selection process is taken in Equation (14). TA can verify the credentials of the GH vehicle in the selection process. Multiple vehicle data is sent to the TA for the GH selection, and one of the vehicles is selected as the GH randomly. In Equation (15), the vehicle with invalid credentials is discarded in the GH-selection process.

(13) $$\begin{array}{c} R(\mathrm{ValidPseudo})=\forall \;i14\in x14\wedge i15\in x15\;|\;(i14[2]=i15[1]\wedge i14[4]\\ \neq Expire)\to x15^{\prime }:=x15\cup \left\{i15\left[2\right],i15\left[3\right]\right\}. \end{array}$$

(14) $$\begin{array}{c} R(\mathrm{GH}-\mathrm{Selection})=\forall i16\in x16\wedge i17\in x17\wedge i18\in x18\;|\;(i16[1]=i17[1]\wedge i16[2]\\ =TimeExpire)\to Random\left(i18\left[1\right]\right)\wedge x17^{\prime }:=x17\cup \left\{assign\left(i17\left[2\right]\right),i17\left[4\right]\right\}. \end{array}$$

(15) $$\begin{array}{c} R(\mathrm{GHSelectionFail})=\exists \;i19\in x19\wedge i20\in x20\;|\;(i19[1]=i20[1]\wedge \\ i19\left[4\right]=Invalid)\wedge Discard\left(x20^{\prime }:=x20\cup \left\{i20\left[3\right]\right\}\right). \end{array}$$

After the GH selection, the grouping of vehicles is started. The vehicles add a request to GH for joining the group, as given in Equation (16). The transmission range vehicles may take part in joining the group. The request may pass or fail depending on the verification of a vehicle. In Equation (18), the GH will prohibit a vehicle joining if it has invalid certificates or other credentials. If it has a valid certificate, the vehicle is allowed to join the group, as shown in Equation (17). In Equation (19) on joining the group, the GH will provide Group Identity (GID) to the vehicles.

(16) $$\begin{array}{c} R(\mathrm{RequestJoining})=\forall \;i21\in x21\wedge i22\in x22\;|\;(i22[2],i22[3])\\ =InRange)\wedge Request\left(x22^{\prime }:=x22\cup \left\{i21\left[2\right]\right\}\right). \end{array}$$

(17) $$\begin{array}{c} R(\mathrm{RequestPass})=\forall \;i23\in x23\wedge i24\in x24\;|\;(i23[2],i23[2])\wedge (i24[2],\\ i24\left[3\right])\Rightarrow InRange\wedge (x24^{\prime }:=x24\cup \left\{i24\left[4\right]\to Valid\right\}. \end{array}$$

(18) $$\begin{array}{c} R(\mathrm{RequestFail})=\exists \;i25\in x25\wedge i26\in x26\;|\;(i25[1]\in i26[1]\wedge \\ i25\left[4\right]=Invalid)\wedge Discard\left(x26^{\prime }:=x26\cup \left\{i26\left[3\right]\right\}\right). \end{array}$$

(19) $$\begin{array}{c} R(\mathrm{GroupJoining})=\forall \;i27\in x27\wedge i28\in x28\;|\;Compare\{(i27[2],i27[3])\wedge \\ \left(i28\left[3\right],i28\left[4\right]\right)\}\Rightarrow InRange\to AssignGID(x28^{\prime }:=x28\cup \left\{i28\left[2\right]\right\}. \end{array}$$

After group formation, the neighbor function (NF) is used to count the number of vehicles in the group as specified in Equation (20). The NF will calculate the number of transmission range neighbors in the group. The neighbor threshold is checked in Equation (21) whether to go for a simple pseudonym change or virtual pseudonym change process. On satisfying the neighbor threshold, the message ready for the pseudonym change process is circulated in the group, as stated in Equation (22). The pseudonym change notification is broadcast to the group, and all the vehicles will set the flag value to 0 as specified in Equation (23). The success indicator shows that all the vehicles have successfully changed pseudonyms.

(20) $$\begin{array}{c} R(\mathrm{NCount})=\forall \;i29\in x29\wedge i30\in x30\wedge i31\in x31\;|\;(i30[2]\wedge i31[2])\\ \;\in i29\left[2\right]\wedge Calculate\left(i30\left[5\right],i30\left[6\right]\right)\to (x31^{\prime }:=x31\cup \left\{i31\left[3\right],i31\left[4\right]\right\}. \end{array}$$

(21) $$\begin{array}{c} R(\mathrm{ThreshSuccess})=\forall \;i32\in x32\wedge i33\in x33\;|\;(i32[2]=i33[2]\wedge i32[4]\\ =i33\left[3\right])\to Indication(x33^{\prime }:=x33\cup \left\{i33\left[4\right]\right\}. \end{array}$$

(22) $$\begin{array}{c} R(\mathrm{Circulation})=\forall \;i34\in x34\wedge i35\in x35\;|\;(i34[4]=i35[3])\wedge \\ Notify(x35^{\prime }:=x35\cup \left\{i35\left[4\right]\right\}. \end{array}$$

(23) $$\begin{array}{c} R(\mathrm{Updating})=\forall \;i36\in x36\wedge i37\in x37\;|\;(i36[2]=i37[2]\wedge i37[3]\\ =Success)\wedge ChangeFlag(x37^{\prime }:=x37\cup \{i37\left[4,i37\left[5\right]\right\}. \end{array}$$

In Equation (24), it is shown that the number of transmission range vehicles threshold is not satisfied, then the virtual pseudonym change process is started. The selection of a suitable neighbor depends on the distance between vehicles. First, the distance between neighboring vehicles is calculated, as stated in Equation (25). The distance ranges are compared and checked the minimum distance between every two neighbors, as specified in Equation (26). In Equation (27), the parameters are set for the selection of virtualizer in the group. Each vehicle in the group creates two messages with different pseudonyms, velocity ranges, and locations. These messages are broadcast to the group, as shown in Equation (28).

(24) $$\begin{array}{c} R(\mathrm{ThreshFail})=\forall \;i38\in x38\wedge i39\in x39\;|\;(i38[2]=i39[2]\wedge i38[4]\\ =Fail)\to x39^{\prime }:=x39\cup \left\{i39\left[3\right],i39\left[4\right]\right\}. \end{array}$$

(25) $$\begin{array}{c} R(\mathrm{NeighSelection})=\forall \;i40\in x40\wedge i41\in x41\;|\;(i41[1]\in i40[3]=\\ Neighbors)\wedge Calculate\left(x41^{\prime }:=x41\cup \left\{i41\left[3\right]\right\}\right). \end{array}$$

(26) $$\begin{array}{c} R(\mathrm{MinDist})=\forall \;i42\in x42\wedge i43\in x43\;|\;(i42[2]=i43[2]\wedge i42[3]\\ =Minimum)\wedge x43^{\prime }:=x43\cup \left\{i43\left[3\right]\right\}). \end{array}$$

(27) $$\begin{array}{c} R(\mathrm{Virtualizer}-\mathrm{Selection})=\exists \;i44\in x44\wedge i45\in x45\;|\;(i44[2]=i45[2]\wedge \\ i45\left[1\right]\in i44\left[3\right])\to MsgCreation\left(x45^{\prime }:=x45\cup \left\{i45\left[3\right],i45\left[4\right]\right\}\right). \end{array}$$

(28) $$\begin{array}{c} R(\mathrm{Broadcast})=\forall \;i46\in x46\wedge i47\in x47\;|\;(i46[2]=i47[2]\wedge \\ Broadcast\left(i46\left[3\right],i46\left[4\right]\right))\wedge x47^{\prime }:=x47\cup \left\{i47\left[3\right],i47\left[4\right]\right\}. \end{array}$$

On the successful pseudonym update process, every vehicle’s flag value in the group is set to 0, as specified in Equation (29). Now again, the pseudonym validity of a vehicle is monitored. If a vehicle’s pseudonym is about to expire, and the vehicle is currently out of range of a group. In this situation, the group formation protocol is activated again for the pseudonym change process, as specified in Equation (30). The vehicle in the range of a group will reset the flag value to 1; it indicates ready for the pseudonym-changing process again, as stated in Equation (31).

(29) $$\begin{array}{c} R(\mathrm{Flag}-\mathrm{setting})=\forall \;i48\in x48\wedge i49\in x49\wedge i50\in x50\;|\;(i48[4]\\ \wedge i49\left[5\right])=0\to Successful\wedge x50^{\prime }:=x50\cup \left\{i50\left[4\right]\right\}. \end{array}$$

(30) $$\begin{array}{c} R(\mathrm{OutofRange})=\forall \;i51\in x51\wedge i52\in x52\;|\;(i51[2]\Rightarrow OutRange\wedge \\ i51\left[4\right]\Rightarrow Expire)\to x52^{\prime }:=x52\cup \left\{i52\left[1\right],i52\left[2\right],i52\left[3\right]\right\}. \end{array}$$

(31) $$\begin{array}{c} R(\mathrm{PseudoExpiry})=\forall \;i53\in x53\wedge i54\in x54\;|\;(i53[3]=1\wedge i51[4]\\ \Rightarrow Expire)\to Reset\left(x54^{\prime }:=x54\cup \left\{i54\left[5\right],i54\left[6\right]\right\}\right). \end{array}$$

### 5.2. Formal Modeling and Analysis of Attacker Scenario on Pseudonym-Changing Protocol

**Figure 12:**
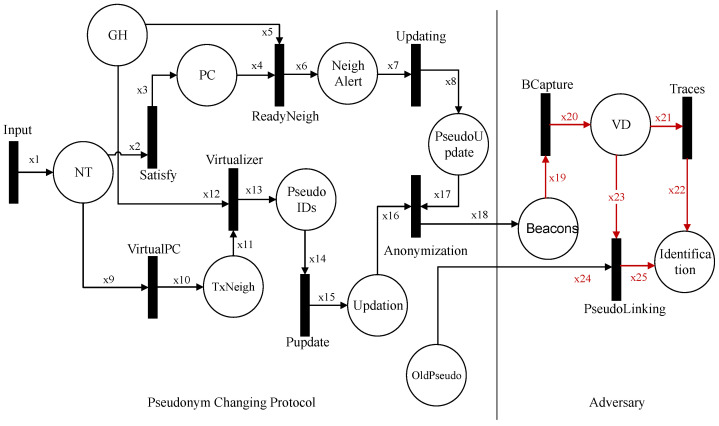
HLPN for adversary analysis on pseudonym-changing protocol.

We formally model and analyze the adversary scenario on the pseudonym-changing protocol. Figure 12 shows the HLPN for the attacker scenario that consists of two entities, i.e., pseudonym-changing protocol and an adversary. The symbols used in HLPN are described in Table 4, while the places used in Petri nets are shown in Table 5. The first transition input is taken from the neighbor function and put into place NT. The input transition contains data about vehicle neighborhoods, such as the number of transmissions range neighbors, the status of vehicle pseudonyms, group information, and group identity. NT in the Petri net takes input the number of neighbors for satisfying the neighbor threshold. In Equation (32), the neighbor threshold is met, and the concerned pseudonym update process is started.

A pseudonym-changing alert is disseminated if vehicles are in the same group (Equation (33)). All vehicles become ready for pseudonym change. In Equation (34), the pseudonym updating process is started, and vehicles change pseudonyms collectively to anonymize pseudo-identities in the group. This creates confusion for an adversary trying to identify the target vehicle.

(32) $$\begin{array}{c} R(\mathrm{Satisfy})=\forall \;i2\in x2\wedge i3\in x3\;|\;(i2[2]=i3[2]\wedge i3[4]=Threshold)\\ \wedge Notify(x3^{\prime }:=x3\cup \left\{i3\left[4\right]\right\}. \end{array}$$

(33) $$\begin{array}{c} R(\mathrm{ReadyNeigh})=\forall \;i4\in x4\wedge i5\in x5\wedge i6\in x6\;|\;(i4[2]\wedge i6[2]\in i5[2]\\ =SameGroup)\to GHAlert(x6^{\prime }:=x6\cup \left\{i6\left[3\right],i6\left[4\right],i6\left[5\right]\right\}. \end{array}$$

(34) $$\begin{array}{c} R(\mathrm{Updating})=\forall \;i7\in x7\wedge i8\in x8\;|\;(i8[1]\wedge i8[2]\in i7[2]\wedge i7[5]=1)\\ \to CollectiveUpdate(x8^{\prime }:=x8\cup \left\{i8\left[3\right],i8\left[4\right]\right\}. \end{array}$$

If the neighbor threshold is not satisfied, then the virtual pseudonym change process is started as specified in Equation (35). The GH selects a few members for a pseudonym update process. The selected members are verified, and each member starts the creation of the messages with different pseudonyms and location information, as stated in Equation (36). Finally, all the selected members of the group take part in the virtual pseudonym change process. The virtual update process is successfully done, as shown in Equation (37).

(35) $$\begin{array}{c} R(\mathrm{VirtualPC})=\forall \;i9\in x9\wedge i10\in x10\;|\;(i10[1]\wedge i10[2]\in i9[2]\wedge i9[3]\\ <i9\left[4\right])\to SelectMembers(x10^{\prime }:=x10\cup \left\{i10\left[3\right],i10\left[4\right],i10\left[4\right]\right\}. \end{array}$$

(36) $$\begin{array}{c} R(\mathrm{Virtualizer})=\forall \;i11\in x11\wedge i12\in x12\wedge i13\in x13\;|\;(i11[2]\wedge i13[2]\\ \in i12\left[2\right])\to Verify\wedge PseudoCreation(x13^{\prime }:=x13\cup \left\{i13\left[3\right],i13\left[4\right]\right\}. \end{array}$$

(37) $$\begin{array}{c} R(\mathrm{Pupdate})=\forall \;i14\in x14\wedge i15\in x15\;|\;(i15[1]\wedge i15[2]\in i14[2]\wedge i14[5]\\ =1)\to VirtualUpdate(x15^{\prime }:=x15\cup \left\{i15\left[3\right],i15\left[4\right],i15\left[5\right]\right\}. \end{array}$$

The primary purpose of both the usual pseudonym update and virtual pseudonym update processes is to anonymize the vehicles in a group. When all the group members change pseudonyms successfully, their pseudo-identities are mixed, making it difficult for an adversary to find a vehicle’s identity and location traces on the road network. Equation (38) shows the anonymization of vehicles after the pseudonym-changing process.

(38) $$\begin{array}{c} R(\mathrm{Anonymization})=\forall \;i16\in x16\wedge i17\in x17\wedge i18\in x18\;|\;(i16[5]\wedge i17[4])=0\\ \to Anonymized\wedge BroadCast(x18^{\prime }:=x18\cup \left\{i18\left[1\right],i18\left[2\right],i18\left[3\right],i18\left[4\right],i18\left[5\right]\right\}). \end{array}$$

After anonymization, each vehicle broadcasts beacons with the new pseudonyms. An adversary has a low-cost transceiver to capture the beacons of vehicles given in Equation (39) and collects vehicle data during the beacons’ broadcast. Based on the vehicle data, the adversary tries to collect two types of information about a vehicle. First, it collects the various location data and tries to match the old location to the vehicle’s new location as specified in Equation (40). Secondly, in Equation (41), the adversary applies a linking attack to link an old pseudonym with a new pseudonym.

(39) $$\begin{array}{c} R(\mathrm{BCapture})=\forall \;i19\in x19\wedge i20\in x20\;|\;(i20[1]\wedge i20[2]\in i19[2])\\ \wedge CollectVD(x20^{\prime }:=x20\cup \left\{i20\left[3\right]\right\}. \end{array}$$

(40) $$\begin{array}{c} R(\mathrm{Traces})=\forall \;i21\in x21\wedge i22\in x22\;|\;(i22[1]\wedge i22[2]\in i21[2])\wedge \\ MatchTraces(x22^{\prime }:=x22\cup \left\{i22\left[4\right],i22\left[5\right]\right\}. \end{array}$$

(41) $$\begin{array}{c} R(\mathrm{PseudoLinking})=\forall \;i23\in x23\wedge i24\in x24\wedge i25\in x25\;|\;(i23[2]=i25[2]\\ \wedge i24\left[2\right]=i25\left[5\right])\wedge Linking\left(i24\left[1\right],i25\left[1\right]\right)\wedge x25^{\prime }:=x25\cup \left\{i25\left[3\right],i25\left[5\right]\right\}. \end{array}$$

The adversary collects vehicle data about location traces and pseudonyms of a vehicle. The pseudonym-changing protocol creates anonymization in the group that increases the confusion of an adversary about the vehicle’s actual pseudonyms. The pseudonym-changing protocol makes it difficult for an adversary to identify a target vehicle in the vicinity.

### 5.3. Formal Modeling and Analysis of Position-Mixing Method

**Figure 13:**
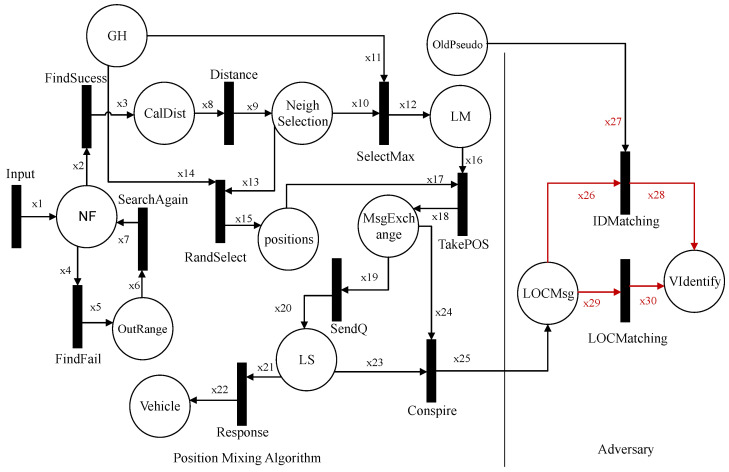
HLPN of position-mixing algorithm.

In this section, we formally model the adversary scenario of the position-mixing method. We design the HLPN of position-mixing algorithm with an attacker scenario shown in Figure 13. The symbols and places used in the HLPN are shown in Table 6 and Table 7, respectively. The HLPN for the position-mixing method takes input transition that consists of data about the vehicle neighborhood, neighbor threshold, and neighboring vehicles’ position coordinates. First, the vehicle will search for a neighbor in the vicinity and select the concerned neighbor as a position mixer.

The neighbor function is used to find transmission range neighbors in the vicinity of a vehicle. Equation (42) shows the success of finding neighboring vehicles and ready to calculate distance among neighboring vehicles. However, the search may be unsuccessful due to the vehicle falling out of range of a group as specified in Equation (43). In Equation (44), the target vehicle will try again to search for vehicles in the transmission range.

(42) $$\begin{array}{c} R(\mathrm{FindSuccess})=\forall \;i2\in x2\wedge i3\in x3\;|\;(i3[1]\wedge i3[2]\in i2[2])\\ \wedge x3^{\prime }:=x3\cup \left\{i3\left[3\right]\right\}. \end{array}$$

(43) $$\begin{array}{c} R(\mathrm{FindFail})=\exists \;i4\in x4\wedge i5\in x5\;|\;(i4[2]\neq i5[2]\wedge i4[3]\neq i5[3])\\ \wedge OutofRange(x5^{\prime }:=x5\cup \left\{i5\left[4\right]\right\}). \end{array}$$

(44) $$\begin{array}{c} R(\mathrm{SearchAgain})=\forall \;i6\in x6\wedge i7\in x7\;|\;(i6[2]\neq i7[2])\to \\ search(x7^{\prime }:=x7\cup \left\{i7\left[2\right],i7\left[3\right]\right\}. \end{array}$$

After finding neighbors in the transmission range, the distance between them is calculated as given in Equation (45). The target vehicle will compare the distance to its neighbors. The neighboring vehicle with maximum distance is selected as a position mixer shown in Equation (46). If the neighbors are in the same distance ranges, then the target vehicle will randomly choose one of the vehicles in its neighbor list specified in Equation (47). In both equations Equations (46) and (47), the selected neighboring vehicle identity is verified with GH’s help.

(45) $$\begin{array}{c} R(\mathrm{Distance})=\forall \;i8\in x8\wedge i9\in x9\;|\;(i9[2]\wedge i9[3]\in i8[2])\wedge \\ Calculate(x9^{\prime }:=x9\cup \left\{i9\left[4\right],i9\left[5\right]\right\}. \end{array}$$

(46) $$\begin{array}{c} R(\mathrm{SelectMax})=\forall \;i10\in x10\wedge i11\in x11\wedge i12\in x12\;|\;(i12[1]=i10[1]\wedge i12[3]=\\ i10\left[2\right])\wedge \left(i12\left[2\right]=i11\left[2\right]\right)\to Verify\wedge x12^{\prime }:=x12\cup \left\{i12\left[4\right],i12\left[5\right]\right\}. \end{array}$$

(47) $$\begin{array}{c} R(\mathrm{RandSelect})=\forall \;i13\in x13\wedge i14\in x14\wedge i15\in x15\;|\;(i15[1]=i13[1]\wedge \\ i15\left[5\right]=i13\left[5\right])\wedge \left(i15\left[2\right]=i14\left[2\right]\right)\to Verify\wedge x15^{\prime }:=x15\cup \left\{i15\left[5\right]\right\}. \end{array}$$

Once the target vehicle selects its position mixer, the position-mixing procedure is invoked. Each vehicle will take its position coordinates and exchange it with its neighboring vehicle chosen, as shown in Equation (48). The position-mixing query is prepared by each vehicle and sent to the location server as specified in Equation (49). On calculating the location query, the location server responds to the vehicle with the desired location, such as the nearest restaurant, gas station, and hospital locations; see Equation (50).

(48) $$\begin{array}{c} R(\mathrm{TakePOS})=\forall \;i16\in x16\wedge i17\in x17\wedge i18\in x18\;|\;(i16[1]\wedge i17[1])\\ \in i18[1]\wedge (i16[3]\wedge i17[3])\in i18[2]\wedge Select(i16[5]ori17[5])\\ \to Exchange(x18^{\prime }:=x18\cup \left\{i18\left[3\right],i18\left[4\right]\right\}). \end{array}$$

(49) $$\begin{array}{c} R(\mathrm{SendQ})=\forall \;i19\in x19\wedge i20\in x20\;|\;(i19[1]=i20[2])\wedge \\ QueryRequest(x20^{\prime }:=x20\cup \left\{i20\left[3\right],i20\left[4\right]\right\}). \end{array}$$

(50) $$\begin{array}{c} R(\mathrm{Response})=\forall \;i21\in x21\wedge i22\in x22\;|\;(i21[1]=i22[1])\wedge \\ QueryResponse(x22^{\prime }:=x22\cup \left\{i22\left[3\right]\right\}). \end{array}$$

We assume that the adversary has the strength to capture location messages communicated to a location server. In Equation (51), the adversary can capture the location messages during communication as well as conspire with the location server to get vehicle location data. The adversary performs two things on location messages, namely, identity matching and location matching attack. In Equation (52), the adversary tries to compare a target vehicle’s identities with different timestamps. However, in Equation (53), the adversary launches a location matching attack on vehicle location data. The adversary tries to match the various location traces of a target vehicle.

(51) $$\begin{array}{c} R(\mathrm{Conspire})=\forall \;i23\in x23\wedge i24\in x24\wedge i25\in x25\;|\;Compare(i23[1],\\ i24\left[1\right],i25\left[1\right])\to Update\left(x25^{\prime }:=x25\cup \left\{i25\left[3\right],i25\left[4\right]\right\}\right). \end{array}$$

(52) $$\begin{array}{c} R(\mathrm{IDMatching})=\forall \;i26\in x26\wedge i27\in x27\wedge i28\in x28\;|\;(i26[1]\wedge i27[1])\\ \in i28\left[1\right]\wedge Match\left(i27\left[2\right],i28\left[2\right]\right)=True\to x28^{\prime }:=x28\cup i28[6]. \end{array}$$

(53) $$\begin{array}{c} R(\mathrm{LOCMatching})=\forall \;i29\in x29\wedge i30\in x30\;|\;(i29[1]=i30[1])\wedge \\ Match\left(i29\left[5\right],i30\left[5\right]\right)=True\to x30^{\prime }:=x30\cup \left\{i30\left[6\right]\right\}). \end{array}$$

The adversary is trying to find the different locations visited by a target vehicle while the position-mixing algorithm hides the adversary’s identities and locations. In the position-mixing method, the location requested messages contain the group identity and location coordinates of a neighboring vehicle that hides and mixes a target vehicle with its neighbor, this producing difficulty for an adversary to identify the target vehicle accurately.

## 6. Experimental Evaluation Setup

The experimental evaluation of our proposed scheme is explained in this section. The first subsection gives a detailed discussion about the simulation setup, and various parameters used for the simulation scenario. In the second subsection, we talk about privacy evaluation metrics in detail.

### 6.1. Simulation Parameters

**Figure 14:**
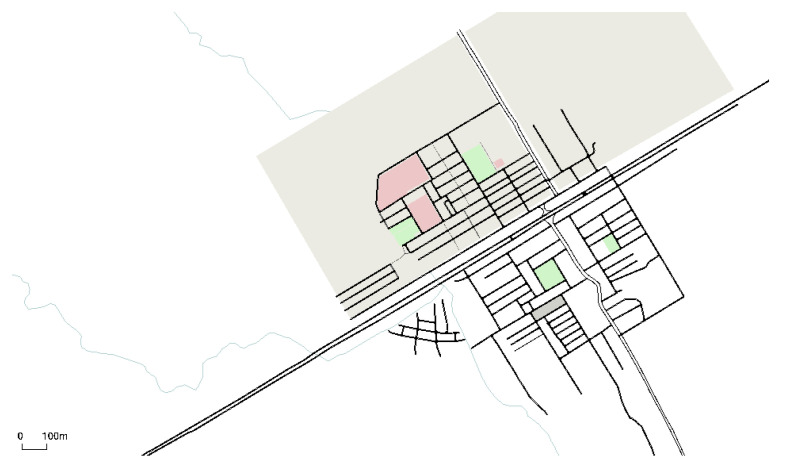
SUMO OpenStreet Map scenario.

We use SUMO for real-world road traffic scenarios. First, the OpenStreet map is used to create a road map of vehicles, as shown in Figure 14. The map is converted to the SUMO network using netconvert and ployconvert tools. The randomTrips python script is used to generate trips for vehicles. Then a vehicle mobility file is created that contains vehicles’ movements on the road network. The proposed scheme is implemented in NS-2. We take 150 vehicles on the road with a speed range between 0–20 m/s. The simulation is run for 300 s. Detail of the simulation parameters are given in Table 8. The results of the proposed scheme are compared with existing schemes CPS [10] and CLPS [18], which are discussed in the coming sections.

### 6.2. Evaluation Metrics

Various metrics are used to evaluate location privacy in a vehicular network. Most of the existing research work considers anonymity set size, entropy, and traceability. The detail of these parameters is given below.

#### 6.2.1. Anonymity Set Size

The Anonymity Set Size (ASS) is used to measure vehicle location privacy in the vehicular communication network. The ASS means the set of indistinguishable vehicles, including the subject or target vehicle [29]. The purpose of ASS is to anonymize the vehicles in the group of vehicles of similar status. The achieved level of vehicle privacy depends on the anonymization process. The higher the anonymization of vehicles, the higher the privacy protection will be. Our proposed scheme DGVP, combines similar status vehicles in a group and changes pseudonyms simultaneously. The cooperation of vehicles for pseudonym-changing is considered a Poisson process with vehicle arrival rate λ, and *X* is the random variable that specifies the number of vehicles in a group at a specific time *T*. The probability of vehicles is computed as given in Equation (54) [46].

(54) $$\begin{array}{c} P\left(X=x\right)=\frac{{\left(\lambda T\right)}^{x}}{x!}e^{-(\lambda T)}. \end{array}$$

λT the expected number of vehicles that update pseudonyms simultaneously. The expected number of vehicles in a group at time *T* is specified as:

(55) $$\begin{array}{c} E\left(X=x\right)=\sum_{x=1}^{\infty }x\frac{{\left(\lambda T\right)}^{x}}{x!}e^{-(\lambda T)}=\lambda T. \end{array}$$

The anonymity set of the expected number of vehicles can be calculated as follows:

(56) $$\begin{array}{c} \left|ASS\right|=\sum_{i,j=1}^{n}Veh_{i}Pseudo_{j}=\sum_{i=1}^{n}E(X_{i}=x)=\sum_{j=1}^{n}\lambda_{j}T. \end{array}$$

where $Veh_{i}Pseudo_{j}$ is the number of vehicles that change pseudonyms simultaneously in the group for anonymization of pseudo-identities.

#### 6.2.2. Entropy of ASS

The entropy value measures an adversary’s confusion level to identify a target vehicle in the group of vehicles. It computes the degree of uncertainty of an adversary to link a target vehicle’s pseudonyms at various locations. To calculate the entropy, let us take $V_{i}$ to be a set of vehicles in a group that takes part in the pseudonym-changing process, and $V_{j}$ is the number of vehicles that update pseudonyms successfully in a group. Let $P_{V_{i}}\to_{V_{j}}$ be the uniform probability of distribution that measures the level of entropy and confusion for an adversary. The adversary tries to find a link between the used pseudonyms of a vehicle at different periods. The entropy of vehicles at time *t* can be computed as follows [46].

(57) $$\begin{array}{c} H_{t}=\sum_{V_{i},V_{j}\in V}P_{V_{i}}\to_{V_{j}}log_{2}P_{V_{i}}\to_{V_{j}}. \end{array}$$

where $H_{t}$ is the entropy of vehicles in a group, and *V* is the total number of vehicles. The average entropy $H_{avg}$ is calculated as follows:

(58) $$\begin{array}{c} H_{avg}=\frac{1}{V}\sum_{i,j\in V}H_{t}\left(i,j\right). \end{array}$$

#### 6.2.3. Location Traceability

Location traceability measures the tracking probability of a vehicle being traced at various locations. Traceability is inversely proportional to the level of location privacy. Let $T_{v}$ is the location traceability strength of an adversary to find a vehicle location in a vicinity. Here we measure location traceability in terms of the vehicle anonymity set. The higher the location anonymization, the lower will be the location tracking percentage. $T_{v}$ can be computed as given below [51].

(59) $$\begin{array}{c} T_{v}=[1-Pr(|ASS|)]. \end{array}$$

The probability of vehicle anonymization is denoted by Pr in Equation (59). When $T_{v}$ is equal to 1, the adversary or attacker successfully tracks a vehicle. The vehicle location anonymization procedure reduces the traceability of a vehicle in the concerned region.

## 7. Performance Comparison

**Figure 15:**
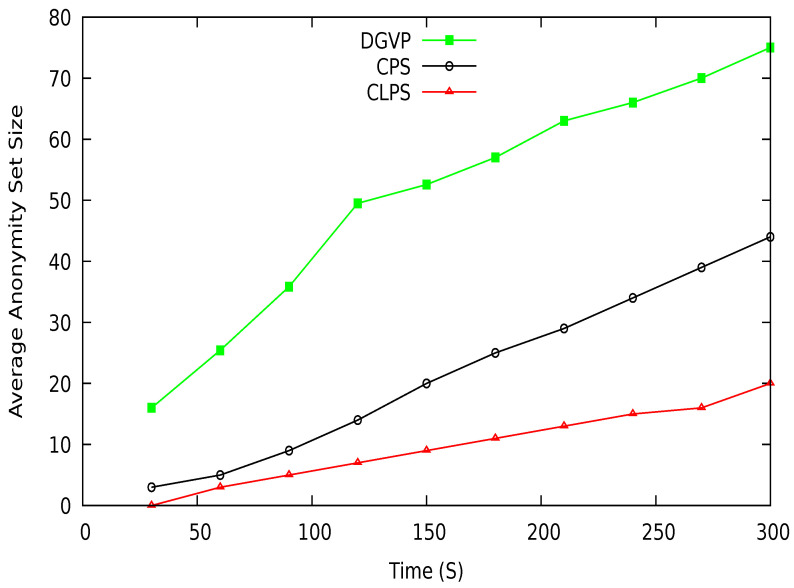
Vehicle anonymization at various time periods.

**Figure 16:**
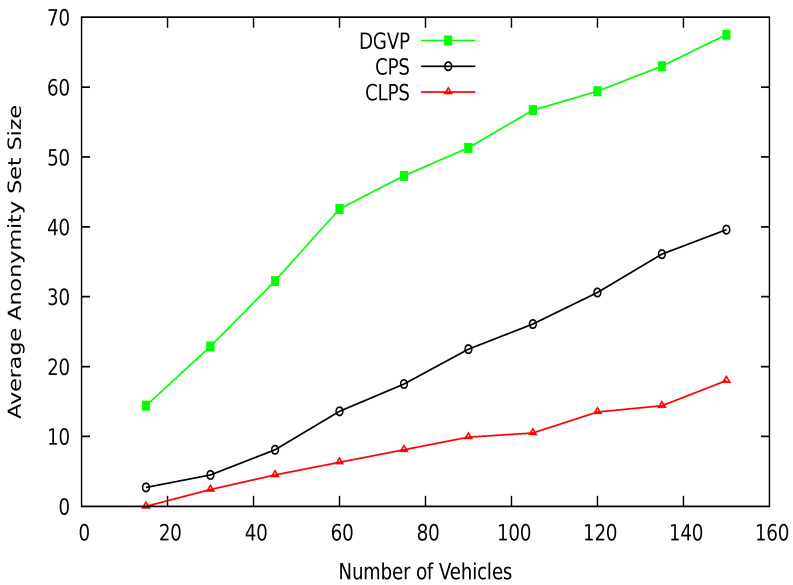
The anonymity of vehicles at different traffic densities.

In this section, we compare the simulation results of our proposed scheme DGVP with Context-based Location Privacy Scheme (CLPS) [18] and Coupling Privacy with Safety (CPS) [10] in terms of anonymity, entropy, and location traceability. The average anonymity set size versus simulation time result is shown in Figure 15. The proposed scheme DGVP improves the anonymization of vehicles in the group compared to CPS and CLPS. The enhanced results of vehicle anonymization is due to the efficient management of vehicles in the group for the pseudonym update process. The non-linearity in the results arises because some vehicles may leave the group’s transmission range or join the group dynamically, reducing or increasing the vehicle anonymization process. Similarly, anonymity versus a different number of vehicles is shown in Figure 16. Here, DGVP increases the anonymity of vehicles to improve the location privacy in the vicinity compared with existing schemes CPS and CLPS. The reason for the lower average anonymity of CLPS is the use of a short pseudonym life, which changes individually by a vehicle before the trigger for collaborative pseudonym change. The reduced level of vehicles’ anonymity in CPS is the lack of cooperation among vehicles for pseudonym change. CPS only considers delay for beacon broadcast but does not ensure the cooperative pseudonym update process in the vicinity, reducing vehicles’ anonymity and privacy.

**Figure 17:**
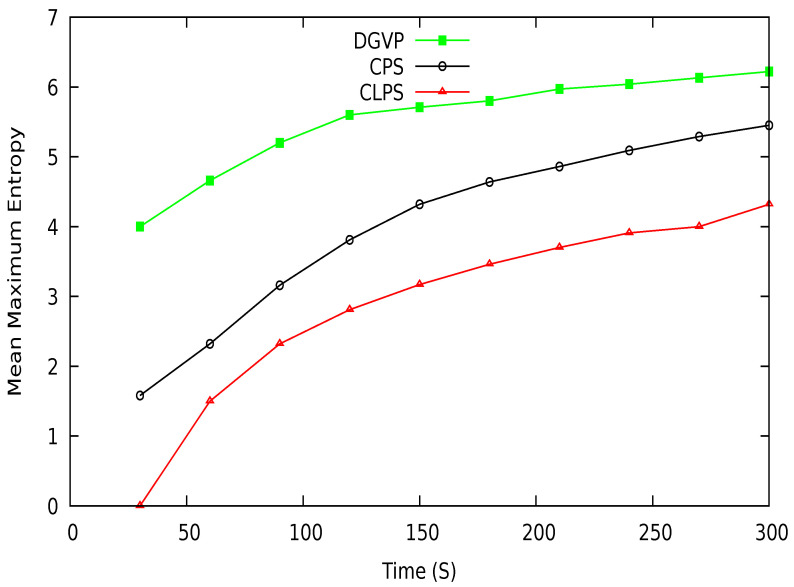
The entropy of vehicles at different periods on the road network.

**Figure 18:**
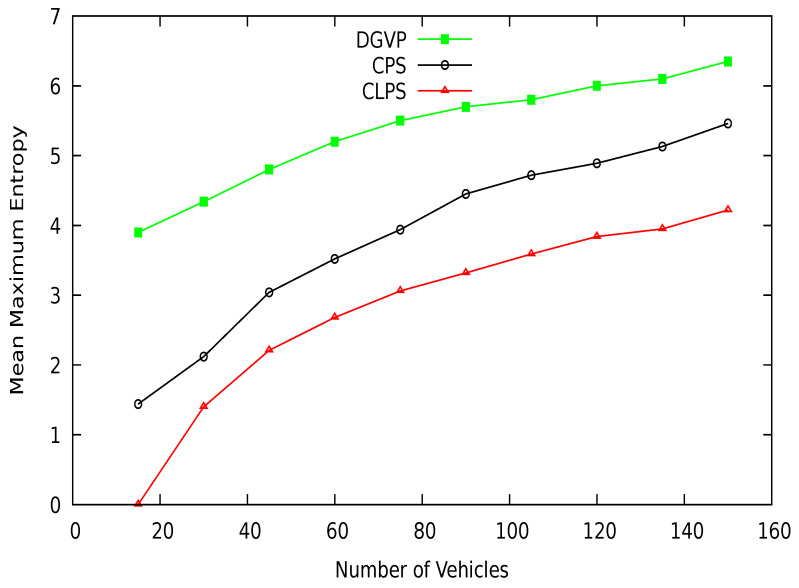
Vehicle entropy at various traffic densities.

Mean maximum entropy in terms of different periods is shown in Figure 17. DGVP achieves better results than existing schemes CPS [10] and CLPS [18]. The entropy measures an adversary’s confusion level about the target vehicle’s identity in the region of interest. DGVP increases confusion for the adversary to identify a vehicle. This is because of the efficient management of anonymization of vehicles in the groups. Similarly, in Figure 18, the results of mean maximum entropy with varying number of vehicles in the vicinity are presented. Our proposed scheme achieves higher confusion for an adversary in extracting the target vehicle in a group than existing CPS and CLPS. It hides the private information of a vehicle from the adversary. The achieved results of a higher level of location privacy is due to the cooperative pseudonym-changing process. The lack of cooperation for pseudonym-changing among vehicles reduces the entropy of CPS. In CLPS, pseudonyms are changed by a smaller number of vehicles in silent mode, ultimately decreasing the mean maximum entropy value.

**Figure 19:**
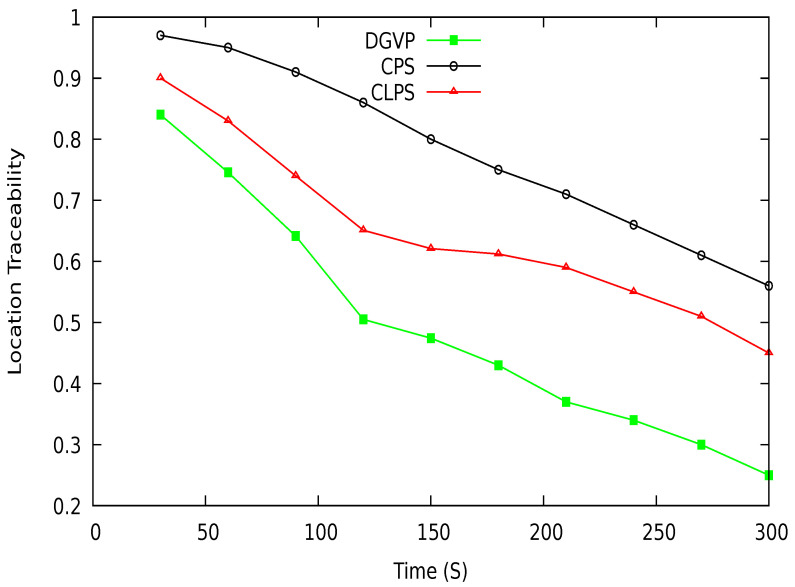
Location traceability at different periods.

**Figure 20:**
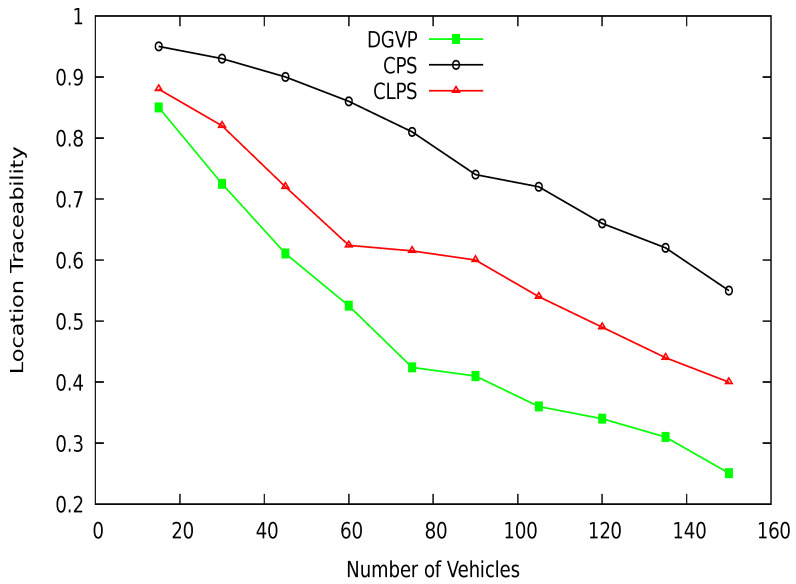
Location traceability at different vehicle traffic conditions.

Figure 19 shows the vehicle location traceability at different periods. At the start of simulations, the traceability probability is higher; after some time, traceability is reduced with the increasing anonymization of vehicles. The proposed scheme DGVP has a lower traceability probability than existing schemes CPS [10] and CLPS [18]. The location traceability percentage with different vehicle traffic conditions is shown in Figure 20. Here also, DGVP has improved results reducing the vehicle location traceability than CPS and CLPS. The efficient results of DGVP are due to the higher value of anonymization of vehicles on the road network. The anonymization process of DGVP increases the difficulty for an adversary to track the vehicle’s various locations during a journey. CLPS has a lower traceability probability than CPS because of the efficient management of silent periods for hiding actual vehicle identity. While in the CPS scheme, the reduced number of beacon broadcasts and use of a pseudonym for a long period provides more chance for an adversary to track a vehicle.

**Figure 21:**
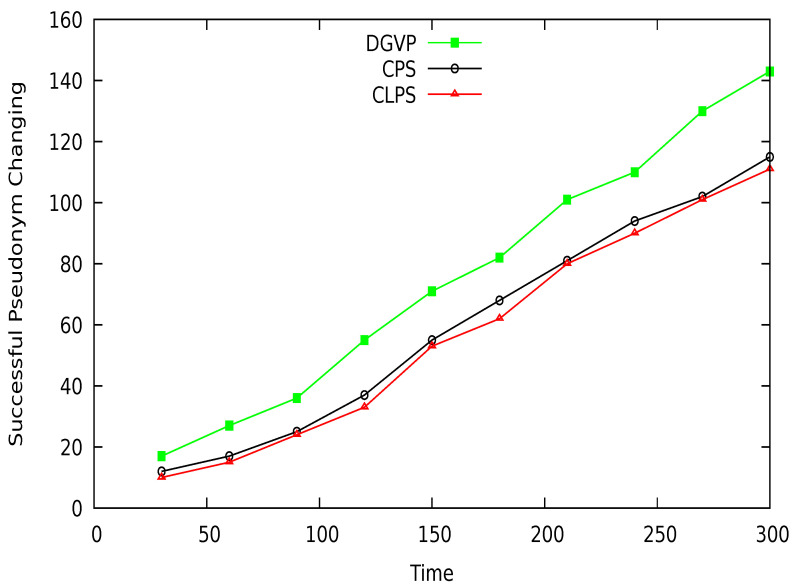
Vehicles successful pseudonym-changing at different time.

The successful changes in pseudonyms of vehicles at different periods are shown in Figure 21. The efficient management of the pseudonym-changing process reduces the possibility of linking a vehicle pseudonym at various locations. The comparative results clearly show that the proposed scheme DGVP outperform CPS [10] and CLPS [18] in terms of successful pseudonym-changing. This is because of the cooperation of neighboring vehicles for the pseudonyms changing process. DGVP manages the grouping of vehicles in the vicinity, providing an environment for vehicles to change pseudo-identities successfully. The lack of cooperation among vehicles in the existing schemes CPS and CLPS reduces the change in pseudo-identities, which increases the possibility for pseudonyms linking attack.

## 8. Position-Mixing Method Results

**Figure 22:**
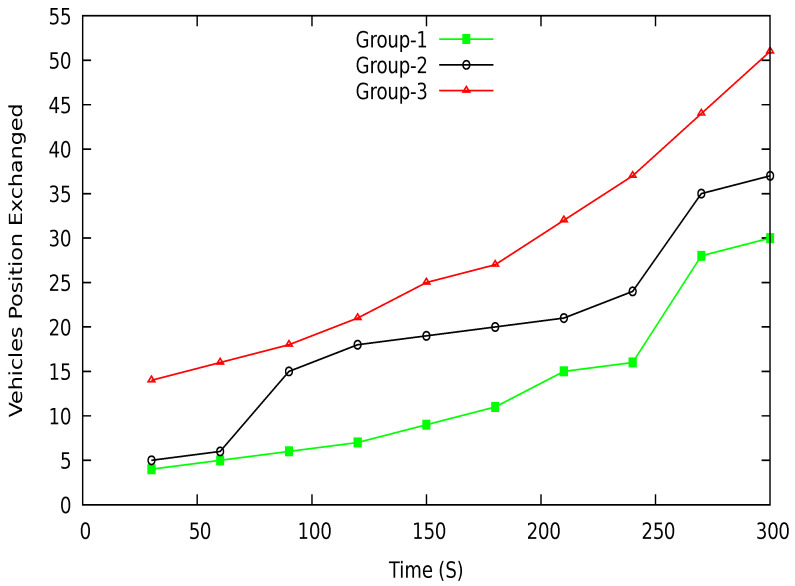
Vehicle position exchanged at different groups.

The vehicle requests LBS for the nearest location of interest; for that purpose, the vehicle shares its position with the location server. We intend to safeguard the location of the vehicle from the third party LBS. In the simulation, we collected the data exchanged with a location server for different groups of vehicles. Figure 22 shows the successful location positions of vehicles exchanged with each other. We take data of three groups to examine the position coordinates exchanged among the vehicles. As shown in the figure, group-1 has a lower number of positions exchanged due to the lower number of vehicles in the group. Group-3 has improved results regarding positions exchanged with neighboring vehicles compared to group-1 and group-2. At different periods, each group has various member vehicles. Over time, the number of vehicles in the group increases, which results in the increased confusion for an adversary to identify a target vehicle in a group. The higher the number of successful positions exchanged in the group, the higher the confusion for an adversary will be.

**Figure 23:**
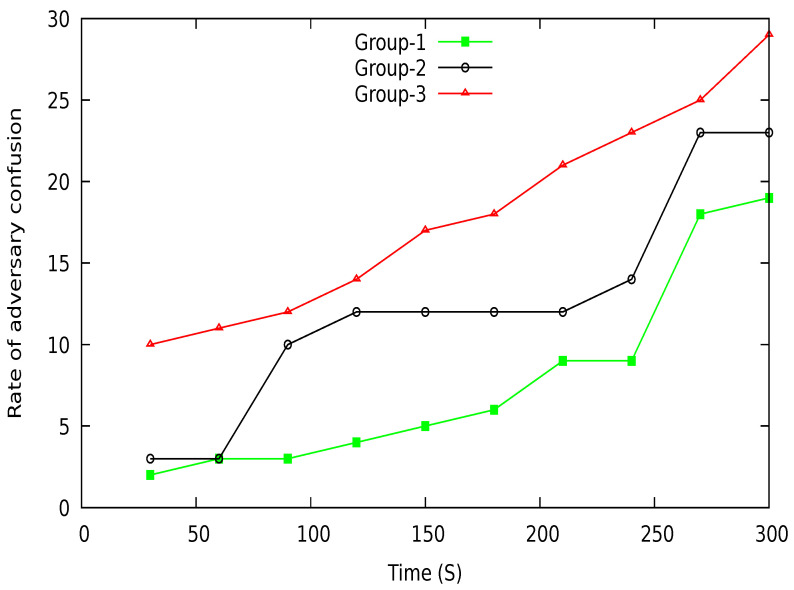
Adversary confusion or ASS with different time spots.

Figure 23 shows the rate of adversary confusion about the actual location of a vehicle taken from the location server or observed during vehicle communication with LBS. Three group results are shown in the figure to analyze the confusion rate of an adversary. The various groups create different uncertainty while exchanging location information with LBS. Each group has a varied number of member vehicles, which affects the confusion rate. Group-3 has a higher number of vehicles than group-1 and group-2, which ultimately increases uncertainty for an adversary to identify the target vehicle. The number of vehicles in the group is time-variant. With time, more vehicles join the group, increasing anonymity and confusion to extract the vehicle’s actual location.

## 9. Analysis and Discussion

We examine the proposed scheme DGVP under various factors; protection against GPA, impact on VANETs applications, time complexity, and computation cost. The detail is given in the following subsections.

### 9.1. Privacy Protection Analysis against GPA

**Figure 24:**
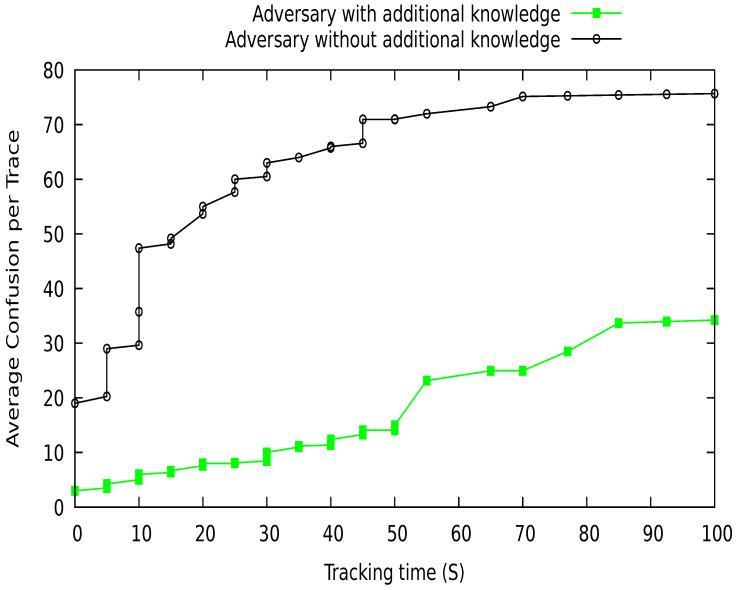
Rate of adversary confusion at various tracking time.

**Figure 25:**
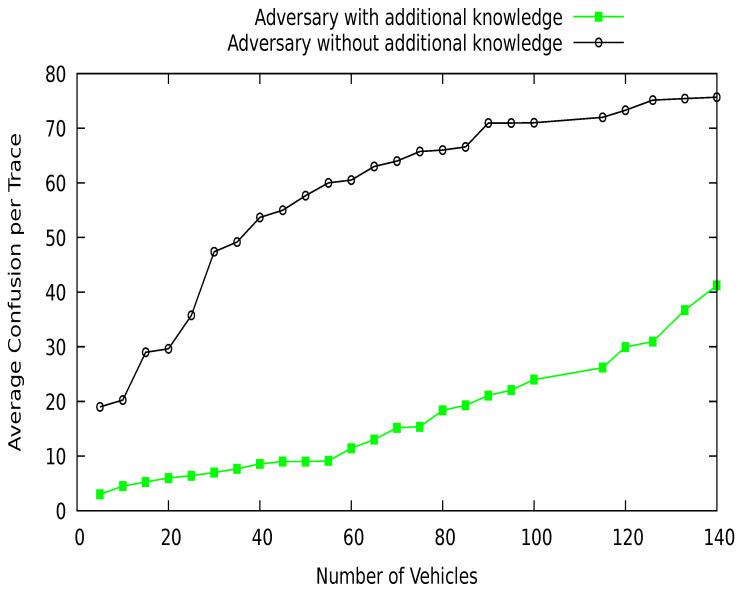
Adversary confusion in identifying vehicles on the road.

In this research, we consider the GPA that uses a low-cost transceiver to eavesdrop the communication among the vehicles on the road. The adversary catches the beacon messages, which contain pseudo-identity and location of vehicles. It tries to match the vehicle pseudo-identities at various locations. Here, we analyze the strength of the adversary (GPA) in terms of with and without additional knowledge. The additional knowledge of adversary about a vehicle may be frequently visited locations, previous pseudonyms, and vehicle locations of interest. This information increases the strength of an adversary to identify a vehicle. The adversary without additional knowledge has no past information about a vehicle; it only tries to explore the recently collected data to identify the target vehicle. We analyze the privacy protection of our proposed scheme DGVP against the strength of an adversary with and without this additional information. Figure 24 shows the average rate of confusion generated for the adversary at various tracking times. The proposed scheme produces higher confusion or uncertainty for adversaries without extra information compared to an advanced adversary. At the start of the procedure, the adversary confusion rate is low; over time, the confusion rate increases for an adversary to link various pseudonyms of a vehicle in the vicinity. So, both adversaries are facing difficulties in tracking vehicles on the road network. Similarly, Figure 25 contains the results of the average confusion per trace in terms of vehicle density. The confusion rate is lower with fewer vehicles. With an increase in vehicle traffic density, the confusion rate increases. Although an advanced adversary has a lower confusion rate than a simple adversary, the proposed scheme still creates uncertainty for an adversary to identify a target vehicle in the network.

### 9.2. DGVP Scheme Impact on VANET Applications

The privacy protection mechanism has some impact on VANET applications. The user wants to hide the private information related to his/her location while also wanting to efficiently manage the vehicular network applications. The proposed scheme, DGVP, uses a neighbor’s data to anonymize the identity of the target vehicle. The pseudonyms of vehicles are updated in a grouped manner. Our privacy scheme provides both location and identity protection to the vehicle while considering the smooth function of VANETs applications. We do not change the relative traffic information broadcast during a vehicle journey; however, the vehicles’ meeting areas are explored to obtain an opportunity for protection of location privacy of the target vehicle. The proposed scheme creates confusion for an adversary to identify a vehicle in a group and does not change any information related to VANET applications such as safety and infotainment applications. We take the expectation of an adversary confusion in terms of anonymization of vehicles in a group [35].

(60) $$\begin{array}{c} E\left(Anonymity\right)=\frac{1}{MN}\sum_{i=1}^{M}\sum_{t=1}^{N}P_{i}\left(t\right)\left[\left(G_{ID_{i}}\left(t\right),PseudoUpdate_{i}\left(t\right)\right)V_{i}\right]. \end{array}$$

where $P_{i}\left(t\right)$ is the probability of an adversary to identify a target vehicle in the group. $G_{ID}$ is the group identity assigned at the time of group creation, $PseudoUpdate_{i}$ is the vehicle pseudonym-changing during the crowd of vehicles, $V_{i}$ is any vehicle moving on the road, *M* is the number of the vehicles taking part in the anonymization process, and *N* is the observation time of vehicle data in the vicinity. The quality of applications (QoA) can be defined in terms of confusion or uncertainty as given in the following equation:

(61) $$\begin{array}{c} QoA=\frac{1}{MN}\sum_{i=1}^{M}\sum_{t=1}^{N}\left[VPseudo_{i}\left(t\right),G_{ID}\left(Pseudo_{i}\left(t\right),Pseudo_{i}^{\prime }\left(t\right)\right)\right]V_{i}. \end{array}$$

where $VPseudo_{i}\left(t\right)$ is the virtual pseudonym used during a lower number of vehicles in a group, $Pseudo_{i}\left(t\right)$ is the initial pseudonym of vehicle and $Pseudo_{i}^{\prime }\left(t\right)$ is the change in the pseudonym of a vehicle after time *t*. In Equation (61), it is clear that the proposed scheme creates confusion for an adversary to link the various pseudonyms of the vehicle at different periods. However, the parameters used for the anonymization of vehicles did not contain any distortion data that reduces vehicular application service quality. Hence, the proposed scheme has a lower impact on the quality of VANET applications.

### 9.3. Algorithm Complexity

We analyze the complexity of algorithms used in the proposed solution in terms of time. Time complexity is the time taken by algorithms to perform a specific task. Our proposed solution has three main algorithms, i.e., vehicle grouping, pseudonym-changing protocol, and position-mixing method. The reason to calculate the time complexity of algorithms is to identify the proposed solution’s total running time concerning privacy protection. Higher time requirements for privacy scheme certainly affects other services of the VANETs such safety services and infotainment services. The complexity of the algorithms is given below.

#### 9.3.1. Vehicle Grouping Algorithm Complexity

To find the vehicle group algorithm’s time complexity, we must consider the GH-selection process, neighbor selection process, and verification of vehicles to join the group. Let $V_{n}$ is the number of vehicles in the vicinity that takes part in the GH-selection process. The cost of computation of selection of GH takes $O(V_{n})$. The neighbor function counts the number of transmission vehicles in the group vicinity. Let $D_{n}$ is the distance calculation among neighboring vehicles in the group, then time complexity for the neighbor function is $O(D_{n}*V_{n})$. After this, the GH starts the joining of transmission range vehicles in the group. GH verify each vehicle’s authenticity, let $VF_{n}$ is the cost of the verification process, then the cost of vehicle verification is $O(VF_{n}*V_{n})$. The time complexity of the vehicle grouping algorithm is given below.

(62) $$\begin{array}{c} Time\;complexity\left(Grouping\right)=O\left(V_{n}\right)+O\left(D_{n}*V_{n}\right)+O\left(VF_{n}*V_{n}\right)\\ =O\left(1+D_{n}+VF_{n}\right)V_{n}\\ =O\left(D_{n}+VF_{n}\right)V_{n}\\ =O(D+VF)V(n)\\ =O(n) \end{array}$$

#### 9.3.2. Pseudonym-Changing Protocol Complexity

The pseudonym-changing protocol takes various road network parameters to update vehicles pseudonyms in a group manner. For the pseudonym-changing algorithm’s complexity, we should consider the selection of virtualizer, messages creation, and pseudonym update process. Let $D_{n}$ be the distance calculation time between GH and group member and $V_{n}$ be the number of vehicles taking part in the virtualizing process; the time complexity of the vehicle virtualizing selection process is $O(D_{n}*V_{n})$. The selected vehicles will create duplicate messages with virtual pseudonyms. Let $MSG_{n}$ be the number of messages designed for the virtual pseudonym-changing process and $V_{n}$ be the vehicles that make duplicate messages, then the computation complexity of messages creation is $O(MSG_{n}*V_{n})$. Now all the group members cooperatively update pseudonyms. Let $PID_{n}$ be pseudonyms, and $V_{n}$ is the number of vehicle update pseudonyms. The time complexity of pseudonyms update is $O(PID_{n}*V_{n})$. The overall computation cost of pseudonym-changing protocol is given below.

(63) $$\begin{array}{c} Time\;complexity\left(Pseudo-Update\right)=O\left(D_{n}*V_{n}\right)+O\left(MSG_{n}*V_{n}\right)+O\left(PID_{n}*V_{n}\right)\\ =O\left(D_{n}+MSG_{n}+PID_{n}\right)V_{n}\\ =O(D+MSG+PID)V(n)\\ =O(n) \end{array}$$

#### 9.3.3. Position-Mixing Algorithm Complexity

The position-mixing algorithm computation complexity consists of the position mixer selection and position exchange process between the vehicles. Let $D_{n}$ be the distance calculation cost between vehicles and $V_{n}$ be the number of vehicles taking part in this process. The computation cost of the position mixer selection process is $O(D_{n}*V_{n})$. After the selection of the position mixer, the vehicle starts to exchange position coordinates with each other. Let $PS_{n}$ be the position coordinates exchange between vehicles $V_{n}$, then the time complexity for the position exchange process is $O(PS_{n}*V_{n})$. The overall time complexity of the position-mixing algorithm is given below.

(64) $$\begin{array}{c} Time\;complexity\left(Position\;mixing\right)=O\left(D_{n}*V_{n}\right)+O\left(PS_{n}*V_{n}\right)\\ =O\left(D_{n}+PS_{n}\right)V_{n}\\ =O(D+PS)V(n)\\ =O(n) \end{array}$$

### 9.4. Cost of Computation

**Figure 26:**
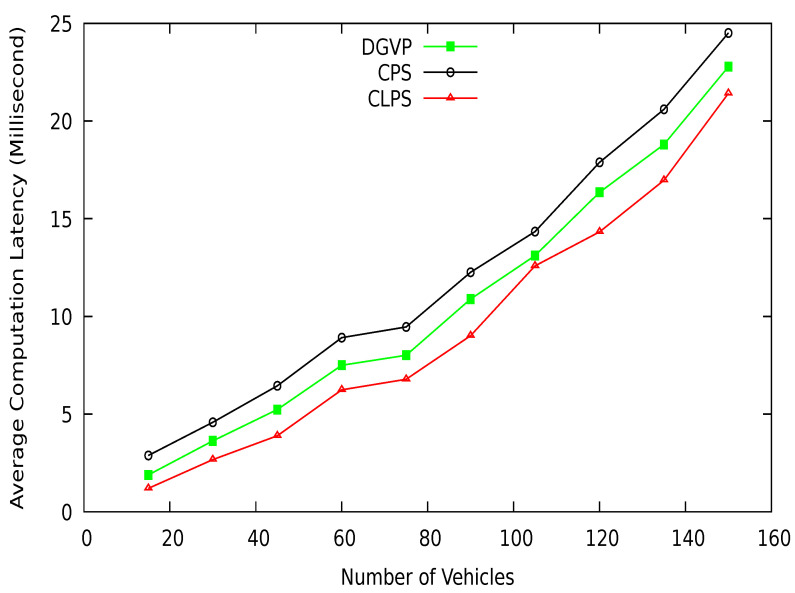
Computation Latency (MS) at different vehicle traffic density.

**Figure 27:**
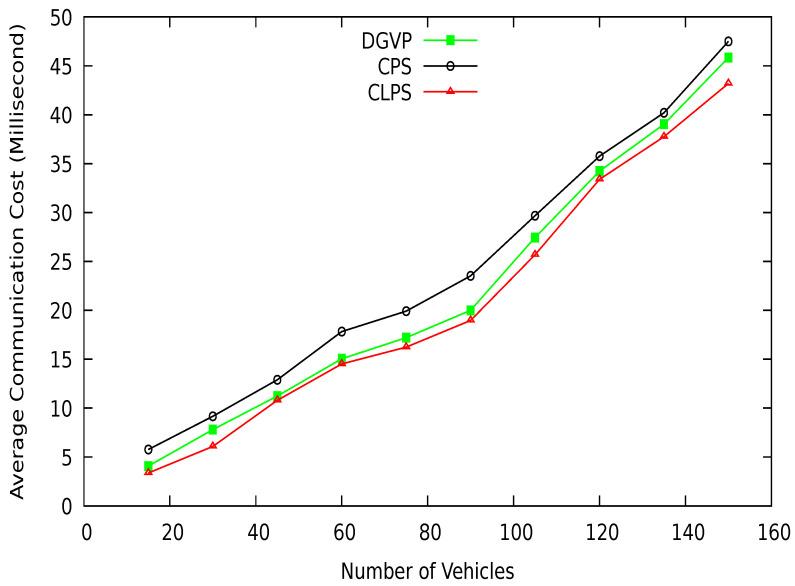
Communication latency at a different number of vehicles.

The cost of computation includes computation latency and vehicle communication latency. The computation latency consists of the time required for group formation, vehicle joining, and other related computations for applying privacy schemes. The communication latency is the time taken by vehicles to communicate with each other for setting up the privacy protection scheme. Figure 26 shows the comparative results of the proposed scheme DGVP and existing schemes CPS and CLPS regarding average computation cost. The CPS requires extra time for distance calculation with RSU, trip time calculation, and pseudonym calculation, which make its computation cost higher than both DGVP and CLPS. The CLPS has a lower computation due to the reduced number of cooperative vehicles in the region. DGVP has modest increase in computation time but provides robust privacy protection compared with CPS and CLPS. The average communication latency at different traffic densities is shown in Figure 27. The communication cost of the proposed scheme DGVP is higher than CLPS but lower than CPS. This is because of communication among the vehicles for the virtual pseudonym process and group communication. The cost of communication of CPS is high due to the communication of vehicles with road infrastructure as well as among the vehicles for the anonymization process. Our proposed scheme achieves improved results regarding vehicle location privacy protection at the cost of a small increase in computation and communication latency.

### 9.5. Discussion

The protection of location privacy in case of vehicles moving at high speed and lower-traffic conditions is a challenging one. In the existing schemes [10,18], it is difficult to provide a high level of location privacy in diverse vehicle traffic, negatively impact on road network applications and the privacy is provided only inside the communication range of an RSU. We have analyzed the proposed scheme DGVP regarding privacy protection, quality of service, impact on VANET applications, and computation cost. Based on the simulation results, DGVP has improved location privacy protection results compared with [10,18]. The time complexity requires the time taken by components of the proposed scheme such as vehicle grouping, pseudonym update process, and position-mixing algorithm to apply privacy protection mechanism [52]. We calculated the time complexity of the proposed algorithm that shows the running time of these algorithms. The provision of security creates some cost [53]. Our proposed scheme, DGVP, has a slightly increased cost of computation. This increase in the cost of the DGVP is due to the communication and computation of the pseudonym update process and cooperation among neighboring vehicles. Still, overall, the proposed scheme achieves a higher level of vehicle location privacy protection in diverse vehicle traffic. The quality of the VANET applications is not compromised in the case of DGVP because there is no distortion of location data in the proposed scheme. The existing schemes [10,18] utilize a silent period that affects road safety applications.

## 10. Conclusions

We have proposed a Dynamic Grouping and Virtual Pseudonym (DGVP) changing scheme to protect location privacy in the case of vehicular communication networks. Road context information, such as vehicle speed, position, and the number of neighboring vehicles, is used to form a dynamic grouping of vehicles. The pseudonym update process takes place to change the pseudonyms of vehicles in a grouped manner. In the case of a lower number of vehicles within transmission range, a virtual pseudonym-changing procedure is used. In the virtual pseudonym change method, some randomized version of the pseudonym is created to anonymize the vehicles in the group. We use the position-mixing method to hide the vehicle’s position and identity while communicating with LBS. The DGVP scheme is formally modeled and specified using HLPN. The formal model shows the correctness of the proposed method. The proposed scheme is validated with the help of simulation results with improved anonymity, entropy, reduced location traceability, lower computation cost, and impact on VANET applications. In the future, we are planning to do more experiments on the diverse nature of the road network to explore other parameters for a vehicle’s location privacy.

## Figures and Tables

**Table 2:** Symbols used in HLPN for DGVP scheme.

Symbol	Description
DistCalculation	Calculation of distance between vehicles
Discard-Request	Discard-request of a Vehicle with invalid credentials
GH	Group Head
Issue-Cert	Issuance certificate to vehicles
LPN	License Plate Number
NF	Neighbor Function
Notify	Notification in the group for pseudonym change
NT	Neighbor Threshold
OBU	On-Board Unit
PExpiry	Vehicle Pseudonym expiration
Pupdate	Pseudonym update process
PseudoValidity	The validity of Pseudonym for some time
RC	Ready for pseudonym change
Reg-Request	Request for vehicle registration to TA
Req-Certificate	Vehicle request for certification
ValidCert	Valid certificate
VPC	The virtual pseudonym change process
VRD	Vehicle registration data
TA	Trusted Authority

**Table 3:** Places used in HLPN for DGVP scheme.

Symbol	Description
φ (Reg-Request)	$P(V_{ID}\times LPN)$
φ (TA)	$P(V_{ID}\times LPN\times PU_{i,k}\times PR_{i,k})$
φ (VRD)	$P(V_{ID}\times LPN\times Vcredentials)$
φ (OBU)	$P(V_{ID}\times P_{ID}\times PU_{i,k}\times P\times CertExpire)$
φ (Discard-Request)	$P(V_{ID}\times LPN\times InvalidCredentials)$
φ (Req-Certificate)	$P(V_{ID}\times LPN\times ExpireCert\times RequireCert)$
φ (PExpiry)	$P(P_{ID}\times PTLimit\times Flag\times Certificate)$
φ (GH)	$P(P_{ID}\times G_{ID}\times LOC\times Flag)$
φ (Grouping)	$P(P_{ID}\times VLR\times T_{x}\times Certificate)$
φ (ValidCert)	$P(P_{ID}\times VLR\times T_{x}\times ValidCredentials)$
φ (JoinGroup)	$P(P_{ID}\times G_{ID}\times VLR\times T_{x})$
φ (NF)	$P(P_{ID}\times G_{ID}\times VLR\times T_{x}\times Dist\times Count)$
φ (NT)	$P(P_{ID}\times G_{ID}\times Count\times Thresh)$
φ (RC)	$P(P_{ID}\times G_{ID}\times Thresh\times Indicator)$
φ (Notify)	$P(P_{ID}\times G_{ID}\times Indicator\times AlertPC)$
φ (PUpdate)	$P(P_{ID}\times G_{ID}\times IndicatorS\times CP\times Flag)$
φ (VPC)	$P(P_{ID}\times G_{ID}\times T_{x}\times SelectNeigh)$
φ (DistCalculation)	$P(P_{ID}\times G_{ID}\times Dist\left(V_{i}\times V_{j}\right))$
φ (Virtualizer)	$P(P_{ID}\times G_{ID}\times MinDist)$
φ (MessagesCreation)	$P(P_{ID}\times G_{ID}\times MSG_{1}\times MSG_{2})$
φ (Pseudo-Update)	$P(P_{ID}\times G_{ID}\times CP\times Flag)$
φ (PsuedoValidity)	$P(P_{ID}\times T_{x}\times Flag\times TLimit)$

**Table 4:** Symbols used in HLPN for attacker scenario in DGVP scheme.

Symbol	Description
BCapture	Capturing of beacon messages
CP	Change pseudonym
Identification	Identification of a vehicle by an adversary
NT	Neighbor threshold
NeighAlert	Alert neighbors for the pseudonym change process
$NP_{ID}$	New pseudo-identity
OldPseudo	Old pseudonyms of a vehicle
PCC	Pseudonym change collectively
PC	Pseudonym change
Pseudo-IDs	Vehicle pseudo-identities
ReadyNeigh	Neighbor vehicles ready for pseudonym change
TS	Timestamp
TxNeigh	Transmission range neighbors
VD	Vehicle data
VirtualPC	Virtual pseudonyms change process

**Table 5:** Places used in HLPN for attacker scenario in DGVP scheme.

Symbol	Description
φ (NT)	$P(P_{ID}\times G_{ID}\times Count\times Thresh)$
φ (PC)	$P(P_{ID}\times G_{ID}\times Thresh\times CPC)$
φ (GH)	$P(P_{ID}\times G_{ID}\times LOC\times Flag)$
φ (NeighAlert)	$P(P_{ID}\times G_{ID}\times Indicator\times AlertPC\times Flag)$
φ (Pseudo-Update)	$P(P_{ID}\times G_{ID}\times PCC\times Flag)$
φ (TxNeigh)	$P(P_{ID}\times G_{ID}\times T_{x}\times NS\times Dist\left(V_{i}\times V_{j}\right))$
φ (Pseudo-IDs)	$P(P_{ID}\times G_{ID}\times MSG_{1}\times MSG_{2})$
φ (Updation)	$P(P_{ID}\times G_{ID}\times SkipVP\times CP\times Flag)$
φ (Beacons)	$P(NP_{ID}\times G_{ID}\times LOC\times S\times D)$
φ (VD)	$P(NP_{ID}\times G_{ID}\times collect\left(P_{ID},LOC\right))$
φ (Identification)	$P(NP_{ID}\times G_{ID}\times OldP_{ID}\times OldTraces\times CLOC)$
φ (OldPseudo)	$P(OldP_{ID}\times LOC\times TS)$

**Table 6:** Symbols used in HLPN for position-mixing method.

Symbol	Description
CalDist	Distance calculation with neighbor vehicles
IDMatching	Linking of vehicle identities used for various timestamp
LM	Location mixer
LOCMsg	Vehicle location messages
LOCMatching	Matching visited locations of a vehicle
LS	Location Server
MaxDist	Maximum distance range with a $T_{x}$ neighbor
NeighSelection	Selection of neighbor vehicle as a position mixer
NF	Neighbor Function
OutRange	The vehicle that gets out of the transmission range
RandSelect	Random selection of neighbor vehicle
SameDist	$T_{x}$ vehicle are in the same distance range
SelectMax	Select a neighbor with a maximum distance range
SendQ	Sending of query to LS for location finding
TakePOS	Taking of position coordinates
VLD	Vehicle location data
VIdentity	Vehicle identity

**Table 7:** Places used in HLPN for position-mixing method.

Symbol	Description
φ (NF)	$P(G_{ID}\times T_{x}\times VL_{R}\times D)$
φ (CalDist)	$P(G_{ID}\times T_{x}\times Dist\left(V_{i},V_{j}\right))$
φ (OutRange)	$P(G_{ID}\times T_{x}\times VLR\times NotinRange)$
φ (NeighSelection)	$P(G_{ID}\times T_{x}\times VL_{R}\times SameDist\times MaxDist)$
φ (GH)	$P(G_{ID}\times P_{ID}\times Verify)$
φ (LM)	$P(G_{ID}\times P_{ID}\times T_{x}\times MaxDist\times Mixer_{j})$
φ (Positions)	$P(G_{ID}\times P_{ID}\times T_{x}\times SameDist\times RandMixer)$
φ (MsgExchange)	$P(G_{ID}\times T_{x}\times POS_{i}\times POS_{j})$
φ (LS)	$P(G_{ID}\times VL\times MixLOC\times ReqLOC)$
φ (Vehicle)	$P(G_{ID}\times P_{ID}\times InterestLOC)$
φ (LOCMsg)	$P(G_{ID}\times P_{ID}\times VL\times D\times LOC_{i})$
φ (OldPseudo)	$P(G_{ID}\times OldP_{ID}\times LOC\times TS)$
φ (VIdentify)	$P(G_{ID}\times P_{ID}\times VL\times D\times LOC_{i}\times Expose)$

**Table 8:** Simulation parameters for DGVP.

Parameters	Values
Simulatorv	NS2, SUMO
Map	OpenStreetMap
Road area	2522 × 2323 m
Speed Range	0–20 m/s
Simulation Time	300 s
Beacon Interval	300 millisecond
Bit rate	6 Mbps
Number of vehicles	150
Transmission range	300 m
Neighbor Radius	100 m
Radius of the group	300 m
Group life	100 s
Routing protocol	AODV

## Data Availability

Not applicable.
